# Dipolar Order Mapping Based on Spin‐Lock Magnetic Resonance Imaging

**DOI:** 10.1002/nbm.70331

**Published:** 2026-06-17

**Authors:** Zijian Gao, Qianxue Shan, Ziqin Zhou, Ziqiang Yu, Weitian Chen

**Affiliations:** ^1^ Department of Imaging and Interventional Radiology The Chinese University of Hong Kong Hong Kong; ^2^ MR Research Collaboration Siemens Healthineers Ltd. Hong Kong

**Keywords:** dipolar order, inhomogeneous magnetization transfer, spin‐lock

## Abstract

Inhomogeneous magnetization transfer (ihMT) is sensitive to dipolar order associated with motion‐restricted macromolecules and can be characterized by the dipolar relaxation time $T_{1D}$. In this study, we propose a spin‐lock MRI framework for $T_{1D}$ quantification. Specifically, we introduce a $T_{1D}$‐sensitive metric, $\text{RATI}O_{\text{dosl}}$, derived from the distinct relaxation rate $R_{\text{dosl}}$, defined as the difference between dual‐frequency and single‐frequency $R_{1\rho }$ measurements. To enable dual‐frequency spin‐lock acquisition, we developed a dedicated rotary‐echo spin‐lock sequence. Based on this framework, we further estimated $T_{1D}$ and the macromolecular proton fraction (MPF) within a unified acquisition. The proposed method was evaluated using numerical simulations, phantom experiments, and in vivo imaging in the healthy human brain. Simulations demonstrated high sensitivity of $\text{RATI}O_{\text{dosl}}$ to $T_{1D}$ and supported the robustness of the proposed approach under the investigated conditions. Phantom experiments showed measurable ihMT contrast and supported the feasibility of $T_{1D}$ estimation using $\text{RATI}O_{\text{dosl}}$. In vivo experiments demonstrated simultaneous $T_{1D}$ and MPF mapping using only three spin‐lock‐prepared images. Across 10 healthy volunteers, mean white matter $T_{1D}$ values ranged from approximately 3.70 to 4.80 ms. By requiring only three contrast‐prepared images, the proposed technique provides a rapid framework for simultaneous $T_{1D}$ and MPF mapping and may facilitate further investigation of dipolar‐order‐sensitive microstructural imaging in vivo.

## Graphical abstract

To quantify the dipolar order relaxation time $T_{1D}$ using spin‐lock MRI technique, we introduce a $T_{1D}$‐specific ratio, denoted as ${\text{RATIO}}_{\text{dosl}}$. A novel rotary‐echo spin‐lock sequence was developed to enable dual‐frequency spin‐lock acquisition. We established a framework to estimate $T_{1D}$ from ${\text{RATIO}}_{\text{dosl}}$, as well as the macromolecular pool fraction (MPF) map. By requiring only three contrast‐prepared images, this technique provides a promising pathway for robust, rapid, and simultaneous $T_{1D}$ and MPF quantification with fewer confounds.

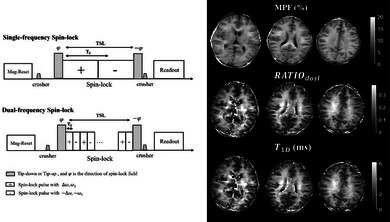

**Abbreviations**
ihMT: inhomogeneous magnetization transfer
MPF: macromolecular proton fraction
MT: magnetization transfer
$R_{1\rho }$
: the spin–lattice relaxation rate in a rotating frame
SL: spin‐lock
$T_{1D}$
: dipolar relaxation time
TSL: time of spin‐lock

## 1 Introduction

In ordered tissues containing motion‐restricted macromolecules, such as myelin, dipolar order exists alongside Zeeman order. This phenomenon manifests in magnetization transfer (MT) experiments as an asymmetric spectrum following single‐frequency saturation, in contrast to the symmetric spectrum observed under dual‐frequency saturation. Known as inhomogeneous magnetization transfer (ihMT), this effect can be quantified by the dipolar relaxation time, $T_{1D}$, and is highly sensitive to tissue microstructure [1, 2, 3, 4, 5]. Consequently, ihMT offers a promising approach for assessing microstructural integrity, particularly brain myelination [6, 7]. It holds significant potential for elucidating disease pathophysiology (e.g., in multiple sclerosis) and providing valuable outcome measures to monitor neuroprotection and repair in clinical trials [8, 9, 10].

The two‐pool model is commonly used to analyze the MT effect, while Provotorov theory, as formulated by Goldman for the dipolar order effect [1], indicates that the MT pool can be subdivided into a Zeeman reservoir and a dipolar reservoir. The parameter β, proportional to the inverse spin temperature of the dipolar reservoir, is incorporated into the two‐pool model [2, 3, 11]. To measure dipolar order under in vivo conditions, Varma et al. introduced ihMT ratio (ihMTR) for in vivo experiments to indicate the ihMT effect via a subtraction experiment between images acquired with single frequency saturation and dual frequency saturation [4]. They further proposed $T_{1D}$ quantification using multiple ihMTR images with varied switch times during dual‐frequency saturation [6]. However, this approach requires a long scan time to acquire sufficient ihMTR images (e.g., eight ihMTR images with different switch times [6]). Prevost et al. proposed an optimized ihMT acquisition strategy based on $T_{1D}$ filtering effect [12], which enables the isolation of short‐ and long‐$T_{1D}$ components by manipulating switch times [13]. Hertanu et al. further showed that the long‐$T_{1D}$ component is highly specific to myelination, whereas short‐$T_{1D}$ components are associated with nonmyelin protons [14]. These findings indicate that multiple ihMTR measurements are needed when the objective is to resolve multiple dipolar components with distinct $T_{1D}$ values. By contrast, under the simplifying assumption of an apparent single long‐$T_{1D}$ component and fixed MT parameters, a single or limited number of ihMT measurements may be sufficient for $T_{1D}$ estimation.

In parallel, a pseudo‐quantitative ihMT method based on inverse‐subtraction metrics has also been developed [9, 15]. This approach enables rapid acquisition with good reproducibility and reduced sensitivity to several confounding factors, and it has been applied in human studies [16, 17, 18]. Malik et al. further proposed a rapid steady‐state pulse sequence for generating ihMT contrast using multiband RF pulses, which simultaneously provide off‐resonance saturation and on‐resonance excitation [19]. Nevertheless, these approaches remain semiquantitative and do not directly quantify dipolar order. More recently, West et al. proposed an MR fingerprinting framework for quantifying $T_{1D}$, macromolecular proton fraction (MPF), and the $T_{1}$ of the free‐water pool [20]. Despite these advances, the development of methods for $T_{1D}$ quantification that can assess myelin microstructural integrity while minimizing confounding effects and maintaining clinically feasible scan times remains highly valuable [8, 21, 22].

Recently, an off‐resonance spin‐lock approach (MPF‐SL) was proposed for quantitative MT [23, 24, 25]. In MPF‐SL, appropriately designed off‐resonance spin‐lock acquisitions are used to remove the free‐water contribution from the rotating‐frame relaxation rate, $R_{1\rho }$. This yields an MT‐specific relaxation rate, $R_{\text{mpfsl}}$, from which MPF can be estimated. Consequently, MPF‐SL allows the use of RF pulses with higher saturation efficiency without exacerbating direct water saturation. By isolating the macromolecular contribution from the water‐pool contribution, this approach substantially simplifies the model for MPF quantification and eliminates the need for prior knowledge or separate estimation of free‐water relaxation times. In addition, the off‐resonance spin‐lock design enables this method to suppress residual dipolar coupling effects in clinical scans [26, 27].

In this study, we extend this approach to quantify the dipolar relaxation time, $T_{1D}$, under the assumption of a single apparent long‐$T_{1D}$ component. With our proposed method, both the MPF and $T_{1D}$ can be acquired in a single fast scan, providing complementary information for tissue assessment. To probe the ihMT effect, we utilize a chain of rotary echo spin‐lock radiofrequency (RF) pulses with variable switch times. These pulses alternate between positive and negative resonance frequency offsets, synchronized with RF phase alterations, to maintain magnetization spin‐locking throughout the pulse duration. These spin‐lock acquisitions eliminate free water contributions from the signal model, thereby substantially simplifying the calculation of $T_{1D}$. Furthermore, MPF is quantified from the same dataset without requiring additional scans. We demonstrate the efficacy of this method using numerical simulations, phantom experiments, and in vivo measurements.

## 2 Theory

In the two‐pool model for MT, tissue magnetization is commonly divided into a water pool ($M_{x}^{a},M_{y}^{a},\text{and}\;M_{z}^{a}$) and an MT pool ($M_{z}^{b}$) [28]. Based on Provotorov theory as formulated by Goldman [1], the MT pool can be further described as comprising both a Zeeman reservoir and a dipolar reservoir. The parameter β, which is proportional to the inverse spin temperature of the dipolar reservoir, is incorporated into the magnetization vector [2, 3, 11]

(1) $$\vec{M}={\left(M_{\mathrm{ax}},M_{\mathrm{ay}},M_{\mathrm{az}},M_{\mathrm{bz}},\beta \right)}^{T},$$

which follows:

(2) $$\frac{d}{\mathrm{dt}}\vec{M}=A\cdot \vec{M}+\vec{C}.$$

Following the notation by Zaiss et al. [29] and incorporating dipolar order [11], we express A as a 5 × 5 system matrix:

(3) $$\begin{array}{c} \begin{array}{c} A=\left(\begin{array}{ccccc} -R_{2a} & -\Delta \omega & 0 & 0 & 0\\ +\Delta \omega & -R_{2a} & +\omega_{1} & 0 & 0\\ 0 & -\omega_{1} & -R_{1a}-k_{\mathrm{ab}} & k_{\mathrm{ba}} & 0\\ 0 & 0 & k_{\mathrm{ab}} & -R_{1b}-R_{\mathrm{rfb}}-k_{\mathrm{ba}} & R_{\mathrm{rfb}}∆\omega \\ 0 & 0 & 0 & R_{\mathrm{rfb}}\frac{∆\omega }{D^{2}} & -\left(\frac{1}{T_{1D}}+R_{\mathrm{rfb}}{\left(\frac{∆\omega }{D}\right)}^{2}\right) \end{array}\right) \end{array} \end{array}$$

and $\vec{C}$ is a constant vector:

(4) $$\vec{C}={\left(0,0,R_{1a}M_{0a},R_{1b}M_{0b},0\right)}^{T}$$

where $T_{1D}$ is the dipolar relaxation time. $R_{2a}$ and $R_{1a}$ are the transverse and longitudinal relaxation rates for the water pool, respectively. $R_{1b}$ is the longitudinal relaxation rate for the MT pool. $R_{\mathrm{rfb}}=\omega_{1}^{2}\mathrm{\pi g}\left(T_{2b},∆\omega \right)$, where $g\left(T_{2b},∆\omega \right)$ denotes the absorption lineshape of the MT pool, and we use a super‐Lorentzian lineshape model in this study [3]. $T_{2b}$ is an MT‐pool parameter that characterizes its absorption lineshape. $M_{0a}$ and $M_{0b}$ denote the equilibrium magnetizations of the water and MT pools, respectively. ∆ω is the resonance frequency offset (FO) and $\omega_{1}$ is the $B_{1}$ amplitude of the spin‐lock RF pulse, or equivalently the frequency of spin‐lock (FSL). $k_{\mathrm{ab}}$ and $k_{\mathrm{ba}}$ are the exchange rates between the water pool and the MT pool. D is associated with the local dipolar field (in angular frequency units) [3], which approximately equals to $\frac{1}{T_{2b}\;\sqrt{15}}$. In addition, we assume the fraction of dipolar order $f_{D}$= 1 [3, 11].

When dual‐frequency RF is applied with simultaneous irradiation at positive and negative frequency offsets, the term proportional to ∆ω in Equation 3 is canceled. It indicates the contribution from dipolar reservoir can be removed using dual‐frequency RF irradiation. Consequently, $A_{\text{dual}}$ simplifies to:

(5) $$A_{\text{dual}}=\left(\begin{array}{cccc} -R_{2a} & -\Delta \omega & 0 & 0\\ +\Delta \omega & -R_{2a} & +\omega_{1} & 0\\ 0 & -\omega_{1} & -R_{1a}-k_{\mathrm{ab}} & +k_{\mathrm{ba}}\\ 0 & 0 & +k_{\mathrm{ab}} & -R_{1b}-R_{\mathrm{rf}b}-k_{\mathrm{ba}} \end{array}\right)$$

In the rotating frame, the relaxation rates $R_{1\rho }$ measured under single‐frequency spin‐lock irradiation ($R_{1\rho }^{\text{single}}$, with $\Delta \omega^{s}$ and $\omega_{1}^{s}$) and dual‐frequency spin‐lock irradiation ($R_{1\rho }^{\text{dual}}$, with $\Delta \omega^{d}$ and $\omega_{1}^{d}$) are primarily determined by the least negative eigenvalue of A and $A_{\text{dual}}$ [29, 30], respectively:

(6) $$R_{1\rho }^{\text{single}}=R_{W}\left({\Delta \omega }^{s},{\omega_{1}}^{s}\right)+R_{\mathrm{MT}}^{s}\left({\Delta \omega }^{s},{\omega_{1}}^{s}\right)$$

and

(7) $$R_{1\rho }^{\text{dual}}=R_{W}\left({\Delta \omega }^{d},{\omega_{1}}^{d}\right)+R_{\mathrm{MT}}^{d}\left({\Delta \omega }^{d},{\omega_{1}}^{d}\right)$$

Here, $R_{W}$ represents the effective relaxation rate of the water pool, whereas $R_{\mathrm{MT}}^{s}$ and $R_{\mathrm{MT}}^{d}$ denote the relaxation rates associated with the MT pool under single‐frequency and dual‐frequency spin‐lock irradiation, respectively. Notably, $R_{1\rho }^{\text{single}}$ contains a contribution from dipolar order, whereas this contribution is suppressed in $R_{1\rho }^{\text{dual}}$. Under the conditions ${\Delta \omega }^{d}\gg {\omega_{1}}^{d}$ and ${\Delta \omega }^{s}\gg {\omega_{1}}^{s}$, the influence of chemical exchange is negligible. When single‐frequency and dual‐frequency spin‐lock are applied with the same direction of spin‐lock field (i.e., $\frac{∆\omega^{d}}{\omega_{1}^{d}}=\frac{∆\omega^{s}}{\omega_{1}^{s}}$), the water pool contribution $R_{W}$ can be removed by taking the difference between $R_{1\rho }^{\text{single}}$ and $R_{1\rho }^{\text{dual}}$. We therefore define a distinct relaxation rate, $R_{\text{dosl}}$, associated with the ihMT effect (see Supporting Information 1 for details):

(8) $$\begin{array}{c} \begin{array}{c} R_{\text{dosl}}=R_{1\rho }^{\text{dual}}-R_{1\rho }^{\text{single}}=R_{\mathrm{MT}}^{d}-R_{\mathrm{MT}}^{s}\\ =f_{b}k_{\mathrm{ba}}\left[\frac{R_{\mathrm{rfb}}^{d}}{k_{\mathrm{ba}}\left(f_{b}+1\right)+R_{\mathrm{rfb}}^{d}}-\frac{R_{\mathrm{rfb}}^{s}}{k_{\mathrm{ba}}\left(f_{b}+1\right)\left(R_{\mathrm{rfb}}^{s}{\left(\frac{∆\omega^{s}}{D}\right)}^{2}T_{1D}+1\right)+R_{\mathrm{rfb}}^{s}}\right] \end{array} \end{array}$$

Here, $R_{\mathrm{rfb}}^{s}$ and $R_{\mathrm{rfb}}^{d}$ correspond to the absorption lineshape terms under single‐frequency and dual‐frequency spin‐lock conditions, respectively. By definition, $k_{\mathrm{ba}}=R\left(1-f_{b}\right)$, where R is the exchange rate. Because $f_{b}$ typically ranges from 0.11 to 0.15 in human white matter [31], implying $f_{b}^{2}\ll 1$, we have $k_{\mathrm{ba}}\left(f_{b}+1\right)=R\left(1-f_{b}^{2}\right)\approx R$. Accordingly, Equation (8) can be simplified to:

(9) $$\begin{array}{c} R_{\text{dosl}}=R_{1\rho }^{\text{dual}}-R_{1\rho }^{\text{single}}=R_{\mathrm{MT}}^{d}-R_{\mathrm{MT}}^{s}\\ =f_{b}R\left(1-f_{b}\right)\left[\frac{R_{\mathrm{rfb}}^{d}}{R+R_{\mathrm{rfb}}^{d}}-\frac{R_{\mathrm{rfb}}^{s}}{R\left(R_{\mathrm{rfb}}^{s}{\left(\frac{∆\omega^{s}}{D}\right)}^{2}T_{1D}+1\right)+R_{\mathrm{rfb}}^{s}}\right] \end{array}$$

To further improve the robustness of $T_{1D}$ estimation, we introduce a variable, ${\text{RATIO}}_{\text{dosl}}$, defined as the ratio of $R_{\text{dosl}}$ measured under two different spin‐lock conditions, denoted $R_{\text{dosl},1}$ and $R_{\text{dosl},2}$, with distinct combinations of Δω and $\omega_{1}$. We impose the following conditions, satisfied in both theoretical derivation and practical implementation:

$$\frac{∆\omega^{d\left(1\right)}}{\omega_{1}^{d\left(1\right)}}=\frac{∆\omega^{d\left(2\right)}}{\omega_{1}^{d\left(2\right)}}=\frac{∆\omega^{s\left(1\right)}}{\omega_{1}^{s\left(1\right)}}>>1,$$

$$∆\omega^{d\left(1\right)}=∆\omega^{s\left(1\right)},$$

and

$$\frac{\omega_{1}^{d\left(1\right)}}{\omega_{1}^{d\left(2\right)}}=\frac{∆\omega^{d\left(1\right)}}{∆\omega^{d\left(2\right)}}=N\;\left(N>1\right).$$

Under these conditions, ${\text{RATIO}}_{\text{dosl}}$ is given by:

(10) $$\begin{array}{c} {\text{RATIO}}_{\text{dosl}}=\frac{R_{\text{dosl},1}}{R_{\text{dosl},2}}=\frac{R_{1\rho }^{\text{dual}\;\left(1\right)}-R_{1\rho }^{\text{single}\;\left(1\right)}}{R_{1\rho }^{\text{dual}\;\left(2\right)}-R_{1\rho }^{\text{single}\;\left(1\right)}}\\ =\left[\frac{R+R_{\mathrm{rfb}}^{d\left(2\right)}}{R+R_{\mathrm{rfb}}^{d\left(1\right)}}*\frac{R_{\mathrm{rfb}}^{d\left(1\right)}R_{\mathrm{rfb}}^{s\left(1\right)}{\left(\frac{∆\omega^{s\left(1\right)}}{D}\right)}^{2}T_{1D}}{R_{\mathrm{rfb}}^{d\left(2\right)}\left(R_{\mathrm{rfb}}^{s\left(1\right)}{\left(\frac{∆\omega^{s\left(1\right)}}{D}\right)}^{2}T_{1D}+1\right)-R_{\mathrm{rfb}}^{s\left(1\right)}}\right] \end{array}$$

In quantitative MT, the exchange rate R and the lineshape parameter $T_{2b}$ are often treated as constants in human studies [32]. Under this assumption, ${\text{RATIO}}_{\text{dosl}}$ is primarily associated with the dipolar relaxation time $T_{1D}$, allowing $T_{1D}$ to be estimated conveniently from ${\text{RATIO}}_{\text{dosl}}$. Notably, MPF can be calculated from $R_{1\rho }^{\text{dual}\left(1\right)}$ and $R_{1\rho }^{\text{dual}\left(2\right)}$ following the method of Hou et al. [23]. This approach removes the confounding influence of dipolar order on MPF estimation without requiring additional data acquisition.

## 3 Method

### 3.1 Acquisition Scheme

**FIGURE 1:**
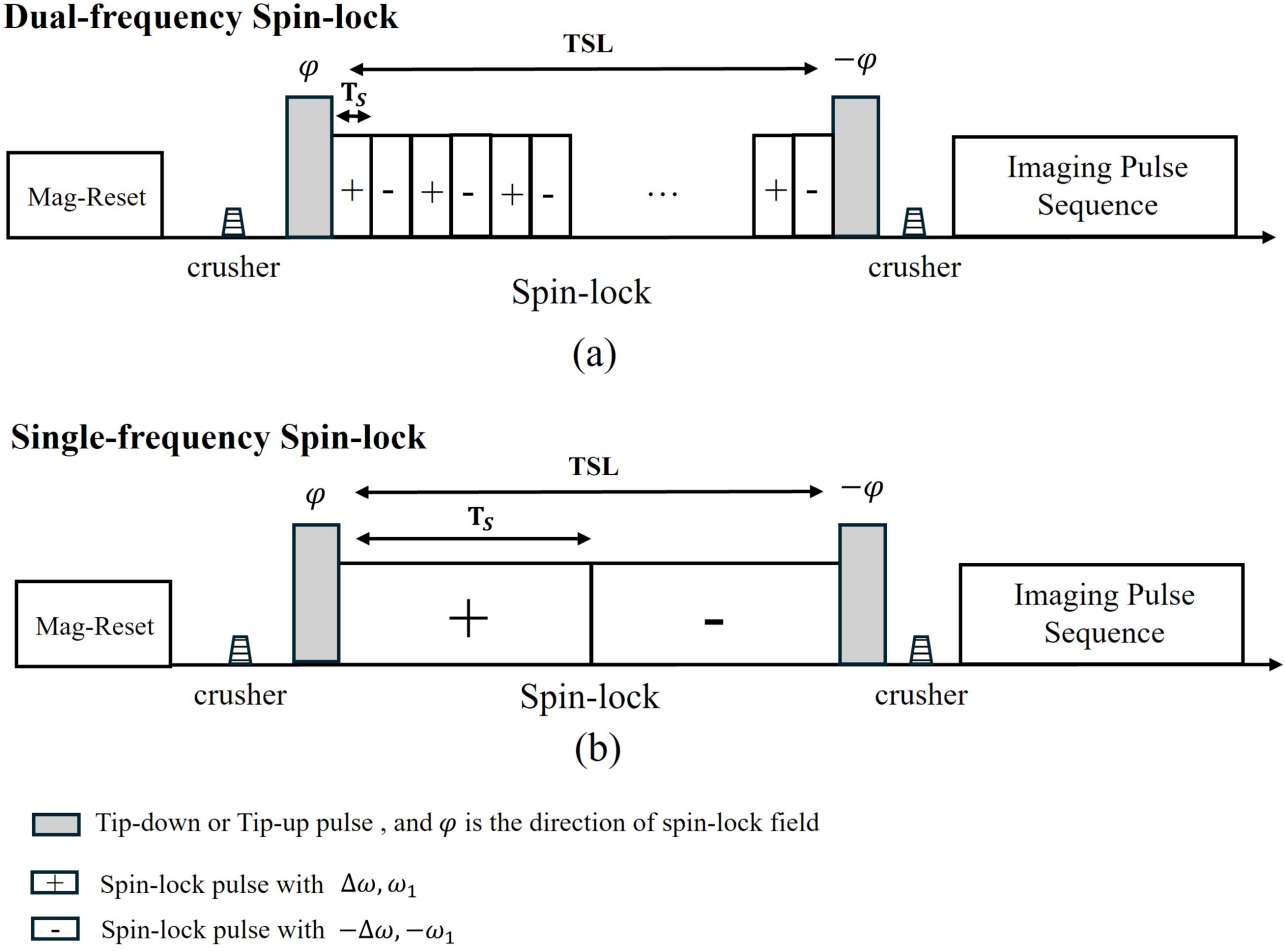
(a) Dual‐frequency spin‐lock pulse sequence with a short switch time $T_{s}$ (shorter than the tissue $T_{1D}$). (b) Single‐frequency spin‐lock pulse sequence with a long switch time $T_{s}$ (longer than the tissue $T_{1D}$).

In existing ihMT acquisition schemes, dual‐frequency saturation can be applied using rapid alternation at positive and negative frequency offsets on a minimal timescale [6]. Similarly, we propose using a chain of rotary echo spin‐lock RF pulses to probe the ihMT effect. In this implementation, the spin‐lock RF pulses alternate between positive and negative resonance frequency offsets with a switching time, $T_{s}$. To maintain magnetization spin‐locking throughout the pulse duration, the RF phase is alternated in synchronization with the frequency offsets. Owing to the $T_{1D}$ filtering effect [13, 14], dual‐frequency spin‐lock is achieved when $T_{s}$ is shorter than the tissue $T_{1D}$ (Figure 1a). In contrast, single‐frequency spin‐lock is implemented using a long $T_{s}$ that considerably exceeds the tissue $T_{1D}$ (Figure 1b). This modified rotary‐echo spin‐lock RF pulse sequence therefore provides a practical method to acquire $R_{1\rho }^{\text{single}}$ and $R_{1\rho }^{\text{dual}}$.

For in vivo experiments, direct measurement of $R_{1\rho }^{\text{single}}$ and $R_{1\rho }^{\text{dual}}$ can be challenging. Robust quantification requires multiple spin‐lock‐prepared images acquired over sufficiently long spin‐lock times (TSLs), which is constrained by specific absorption rate (SAR) and RF hardware limitations. Following the approach reported by Hou et al. [23, 33], we instead acquire data that allow direct calculation of the difference of $R_{1\rho }^{\text{single}}$ and $R_{1\rho }^{\text{dual}}$, rather than measuring the two rates individually. Specifically, $R_{\text{dosl}}=R_{1\rho }^{\text{dual}}-R_{1\rho }^{\text{single}}$ can be obtained from two spin‐lock prepared images using the fast acquisition strategy described by Hou et al. [33]. Consequently, ${\text{RATIO}}_{\text{dosl}}$ can be calculated using a total of only three spin‐lock prepared images:

(11) $$\begin{array}{c} {\text{RATIO}}_{\text{dosl}}=\frac{R_{\text{dosl},1}}{R_{\text{dosl},2}}=\frac{R_{1\rho }^{\text{dual}\;\left(1\right)}-R_{1\rho }^{\text{single}\;\left(1\right)}}{R_{1\rho }^{\text{dual}\;\left(2\right)}-R_{1\rho }^{\text{single}\;\left(1\right)}}\\ =\frac{-\log\left(\frac{M_{d}^{\left(1\right)}-M_{d,\mathrm{ini}}^{\left(1\right)}}{M_{s}^{\left(1\right)}-M_{s,\mathrm{ini}}^{\left(1\right)}}\right)/\mathrm{TSL}}{-\log\left(\frac{M_{d}^{\left(2\right)}-M_{d,\mathrm{ini}}^{\left(2\right)}}{M_{s}^{\left(1\right)}-M_{s,\mathrm{ini}}^{\left(1\right)}}\right)/\mathrm{TSL}}\\ \approx \frac{-\log\left(\frac{M_{d}^{\left(1\right)}}{M_{s}^{\left(1\right)}}\right)/\mathrm{TSL}}{-\log\left(\frac{M_{d}^{\left(2\right)}}{M_{s}^{\left(1\right)}}\right)/\mathrm{TSL}} \end{array}$$

Here, $M_{d}^{\left(1\right)}$ and $M_{d}^{\left(2\right)}$ denote the dual‐frequency spin‐lock prepared images corresponding to relaxation rates $R_{1\rho }^{\text{dual}\;\left(1\right)}$ and $R_{1\rho }^{\text{dual}\;\left(2\right)}$, respectively. $M_{s}^{\left(1\right)}$ denotes the single‐frequency spin‐lock prepared image associated with $R_{1\rho }^{\text{single}\;\left(1\right)}$. The subscript “ini” denotes spin‐lock‐prepared images acquired with a different initial magnetization, which are used to remove the influence of steady‐state magnetization [23]. In brain applications, acquisition of these images can be omitted according to the theory described in the fast acquisition strategy of Hou et al. [33].

### 3.2 Calculation of $T_{1D}$

**FIGURE 2:**
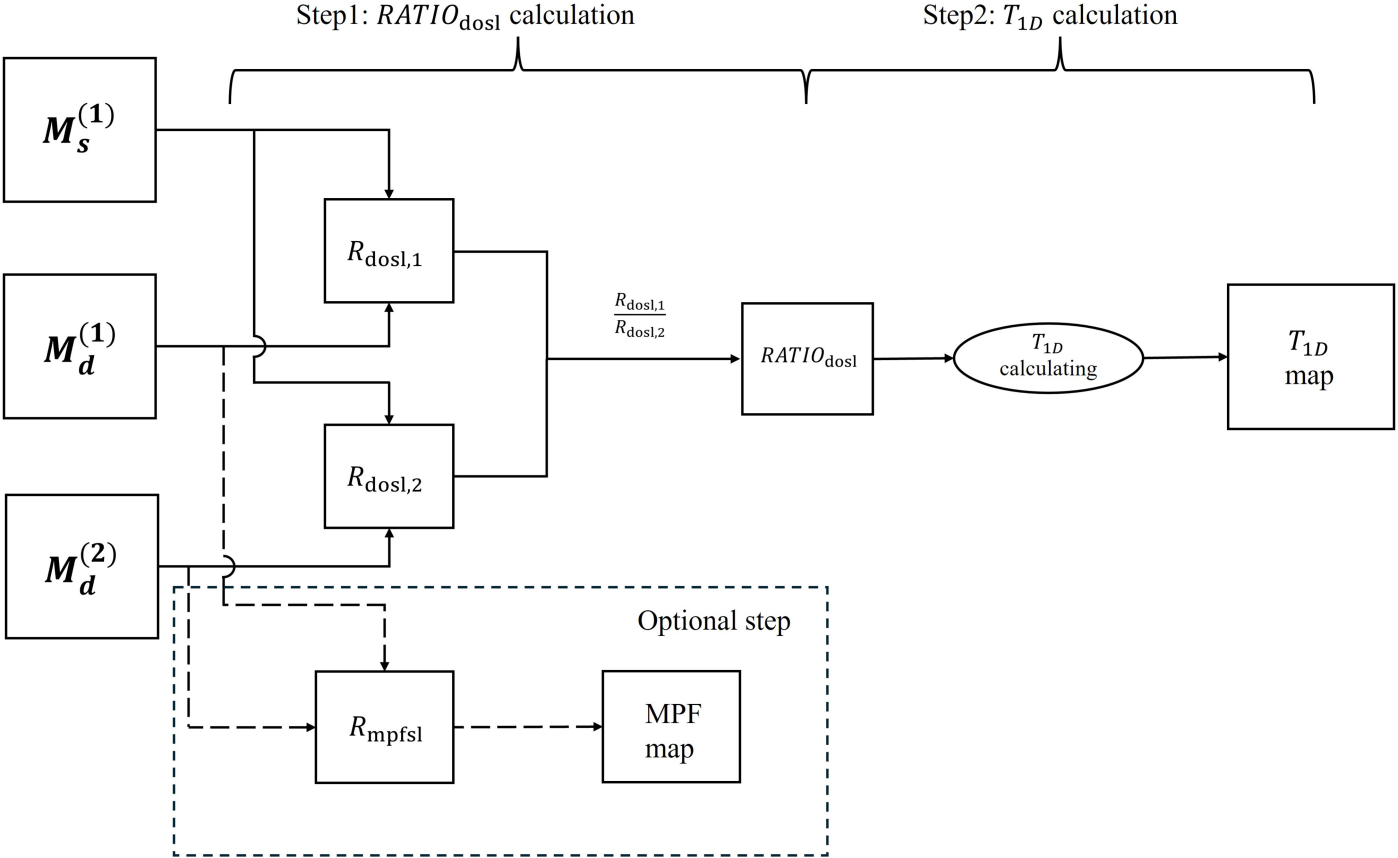
Workflow of the $T_{1D}$ calculation. In Step 1, ${\text{RATIO}}_{\text{dosl}}$ is calculated from three spin‐lock prepared images, $M_{s}^{\left(1\right)}$, $M_{d}^{\left(1\right)}$, and $M_{d}^{\left(2\right)}$. In Step 2, $T_{1D}$ is derived from the ${\text{RATIO}}_{\text{dosl}}$ values. An MPF map can be obtained from $M_{d}^{\left(1\right)}$ and $M_{d}^{\left(2\right)}$ in an optional step [23].

The workflow for calculating $T_{1D}$ consists of two steps, as illustrated in Figure 2. In the first step, ${\text{RATIO}}_{\text{dosl}}$ is computed from $R_{\text{dosl},1}$ and $R_{\text{dosl},2}$ using three off‐resonance spin‐lock prepared images according to Equation 11. In the second step, the $T_{1D}$ map is estimated from ${\text{RATIO}}_{\text{dosl}}$. While multiple numerical approaches exist for this estimation, this study investigates both an analytical estimation and a dictionary‐matching approach. The analytical estimation determines $T_{1D}$ directly by solving Equation 10 using experimentally observed ${\text{RATIO}}_{\text{dosl}}^{\mathrm{acq}}$. In contrast, the dictionary‐matching approach generates a ${\text{RATIO}}_{\text{dosl}}$ dictionary over a predefined range of $T_{1D}$ values; $T_{1D}$ is then determined by matching the measured ${\text{RATIO}}_{\text{dosl}}$ to the corresponding entry in the dictionary.

Under fixed spin‐lock pulse settings and tissue parameters of the two‐pool model, ${\text{RATIO}}_{\text{dosl}}$ is a one‐to‐one function of $T_{1D}$. Accordingly, for each candidate $T_{1D}$, three magnetizations ($M_{d}^{\left(1\right)}$, $M_{d}^{\left(2\right)}$ and $M_{s}^{\left(1\right)}$) are simulated using Bloch‐McConnell‐Provotorov equation (Equation 1–5) following the modified rotary‐echo spin‐lock RF pulses (Figure 1), and the corresponding ${\text{RATIO}}_{\text{dosl}}$ is then derived via Equation 11. The spin‐lock parameters are fixed to match the practical MRI acquisition setup, and tissue parameters of the water pool and the MT pool are held constant according to literature or preacquisition measurements (see Section 3.4.3 for details).

Note the amplitude of RF pulse is incorporated into the lineshape function $R_{\mathrm{rfb}}$. Consequently, to calculate $T_{1D}$ from ${\text{RATIO}}_{\text{dosl}}$, a $B_{1}$ map is acquired to convert the nominal spin‐lock RF amplitude into the actual applied RF amplitude. Given the measured ${\text{RATIO}}_{\text{dosl}}^{\mathrm{acq}}$ and the corresponding local $B_{1}$ value, the optimal entry in the dictionary is identified by minimizing the residual between ${\text{RATIO}}_{\text{dosl}}^{\mathrm{acq}}$ and the simulated ${\text{RATID}}_{\text{dosl}}$. The $T_{1D}$ value associated with this best match is then assigned as the estimated $T_{1D}$.

### 3.3 Simulation Studies

The simulation studies were conducted to validate the proposed theory and the acquisition approach. In all simulation studies, the designed conditions for spin‐lock acquisition parameters were set as: $\frac{∆\omega^{d\left(1\right)}}{\omega_{1}^{d\left(1\right)}}=\frac{∆\omega^{d\left(2\right)}}{\omega_{1}^{d\left(2\right)}}=\frac{∆\omega^{s\left(1\right)}}{\omega_{1}^{s\left(1\right)}}$=10, $∆\omega^{d\left(1\right)}=∆\omega^{s\left(1\right)}$, and $\frac{\omega_{1}^{d\left(1\right)}}{\omega_{1}^{d\left(2\right)}}=\frac{∆\omega^{d\left(1\right)}}{∆\omega^{d\left(2\right)}}=5$. In Simulation study 1, to enable a comprehensive analysis, we vary $\Delta \omega^{d\left(1\right)}$ and $\omega_{1}^{d\left(1\right)}$, and the remaining parameters are adjusted accordingly to satisfy these design conditions. All simulations were performed using custom MATLAB code, which is available on GitHub (https://github.com/zjgao‐spin/Dipolar‐order‐mapping).

#### 3.3.1 Simulation Study 1: Accuracy of Quantification

##### 3.3.1.1 Accuracy of Approximated ${\text{RADIO}}_{\text{dosl}}$

To assess the accuracy of this analytical expression of ${\text{RATIO}}_{\text{dosl}}$ at Equation 10, we compared it against the exact numerical solution of ${\text{RATIO}}_{\text{dosl}}$ obtained by integrating the Bloch‐McConnell‐Provotorov equations. For this comparison, we utilized tissue parameters for white matter derived from previous publications [34, 35]: $T_{1a}=1840\mathrm{ms}$, $T_{1b}=340\mathrm{ms}$, $T_{2a}=69\mathrm{ms}$, $T_{2b}=10\mathrm{\mu s}$, MPF=15%, and $R=23s^{-1}$. We evaluated $T_{1D}$ values of 2, 4, 6, and 8 ms, consistent with the previously reported range for white matter [6, 14]. The ranges for ${\Delta \omega }^{d\left(1\right)}$ and $\omega_{1}^{d\left(1\right)}$ were set to cover common experimental values: 2–7 kHz and 200–800 Hz, respectively. Furthermore, to theoretically validate the sensitivity of the method, we analyzed the relationship between ${\text{RATIO}}_{\text{dosl}}$ and $T_{1D}$ across a range of 1 to 10 ms. Additionally, we investigated how ${\text{RATIO}}_{\text{dosl}}$ varies with other tissue parameters, including $R_{1a}$ (0.3–1.5 Hz), $R_{2a}$ (10–70 Hz), $R_{1b}$ (2–5 Hz), R (15–25 Hz), MPF (10%–20%), and $T_{2b}$ (9–11 μs).

##### 3.3.1.2 Accuracy of $T_{1D}$ Quantification

The analytical estimation does not require generating a dictionary. However, when using this method, the approximate ${\text{RATIO}}_{\text{dosl}}$ model (Equation 10) can introduce bias in the estimated $T_{1D}$ due to underlying theoretical assumptions. This bias between the estimated and ground‐truth $T_{1D}$ can be mitigated by the appropriate selection of acquisition parameters.

To identify parameter regimes that minimize the bias when using the approximate model, we computed the error in the estimated $T_{1D}$ relative to a ground‐truth value of $T_{1D}$= 6.2 ms [6]. Acquired ${\text{RATIO}}_{\text{dosl}}$ values were generated via numerical simulations using Equations 1–5 and 11. The switch times, $T_{s}$, for the dual and single‐frequency spin‐lock pulses were chosen as 0.5 and 40 ms, respectively, given that white matter $T_{1D}$ typically ranges from 3 to 10 ms [7]. The simulation parameters were set as follows: $∆\omega^{d\left(1\right)}/2\pi$ from 2 to 7 kHz, $\omega_{1}^{d\left(1\right)}/2\pi$ ranged from 200 to 800 Hz, TSL from 40 to 120 ms . The $T_{1D}$ values were then calculated by solving Equation 10.

#### 3.3.2 Simulation Study 2: Robustness of the Proposed Method

##### 3.3.2.1 Robustness in Presence of B1 and B0 Inhomogeneity

$B_{1}$ and $B_{0}$ field inhomogeneities are common in MRI systems. We performed simulations to investigate the robustness of the proposed method in the presence of these inhomogeneities. The simulations utilized the same spin‐lock pulse and tissue parameters for white matter as those used in Simulation Study 1, with $\Delta \omega^{d\left(1\right)}/2\pi$, $\omega_{1}^{d\left(1\right)}/2\pi$ and TSL fixed at 5 kHz, 500 Hz and 80 ms, respectively. $B_{1}$ and $B_{0}$ were varied over ranges of 0.7 to 1.3 n.u. and −100 to 100 Hz, respectively. $B_{1}$ and $B_{0}$ inhomogeneities were incorporated throughout the entire spin‐lock preparation, including the tip‐down, spin‐lock, and tip‐up pulses. Note the flip angle used for the tip‐down and tip‐up pulses was $\tan^{-1}\left(\omega_{1}/∆\omega \right)$.

Furthermore, we compared the mean and standard deviation of the estimated $T_{1D}$ with and without $B_{1}$ inhomogeneity correction. In this analysis, $B_{1}$ and $B_{0}$ values were sampled 10,000 times from a uniform distribution over respective range. $T_{1D}$ was estimated using both analytical estimation and dictionary matching. The dictionary was generated covering a $B_{1}$ range of 0.7–1.3 (step size $1\times 10^{-4}$) and a $T_{1D}$ range of 0–15 ms (step size $1\times 10^{-5}$ ms).

##### 3.3.2.2 Robustness Against Variations of the Tissue Parameters

Like other quantitative MT methods, we assumed that certain MT parameters remain constant when calculating $T_{1D}$. ${\text{RATIO}}_{\text{dosl}}$ is predicted to have low sensitivity to most MT parameters, except for $T_{2b}$. In this study, we performed simulations to investigate the errors in the estimation of $T_{1D}$ when MT parameters, specifically $R_{1b}$, R, and MPF, are not constant. We conducted 10,000 simulations with parameters randomly sampled within the following ranges: $R_{1b}=2$–5 Hz, R = 15–25 Hz, and MPF = 10%–20%. We then calculated the variation of the estimated $T_{1D}$ under these parameter variations.

##### 3.3.2.3 Robustness Against Noise

Quantitative imaging requires a sufficient signal‐to‐noise ratio (SNR). In this study, we investigated the influence of SNR on ${\text{RATIO}}_{\text{dosl}}$ and $T_{1D}$ estimation. Simulated MRI signals $M_{d}^{\left(1\right)}$, $M_{d}^{\left(2\right)}$, and $M_{s}^{\left(1\right)}$ were corrupted with additive white Gaussian noise at SNR levels ranging from 20 to 100. For each SNR level, 10,000 random experiments were performed, and the resulting distributions of the estimated $T_{1D}$ were analyzed.

### 3.4 Phantom and In Vivo Studies

#### 3.4.1 Preparation of Phantoms and Health Volunteer

Agar phantoms and Prolipid 161 (PL161; Ashland Specialty Ingredients, USA) phantoms were prepared for this study and underwent the same MRI protocol. The agar phantoms were prepared at 1%, 2%, 3%, and 4% (w/w), while the PL161 phantoms were mixed with pure water at 4%, 8%, 12%, and 16% (w/w). PL161 exhibits strong ihMT contrast and was therefore regarded as a validation of the ihMT effect [5, 20].

Ten healthy volunteers (age range 25–30 years; 5 male and 5 female) were enrolled in this study under the approval of our Institutional Review Board (Ref No. 2016.150). Exclusion criteria included a history of neurological diseases, brain injury, major psychiatric illness, or drug or alcohol misuse. The study was performed in accordance with the institutional ethical guidelines and the ethical standards of the 1964 Declaration of Helsinki and its subsequent amendments. Written informed consent was obtained from all participants. Each volunteer underwent test–retest MRI examinations with a 7–10 day interval.

#### 3.4.2 MRI Protocol

All MRI data acquisitions were performed using a 3T Prisma scanner (Siemens Healthineers, Germany) equipped with a 64‐channel head–neck receiver coil at room temperature (~20°C). The MRI scan protocol for in vivo study included the following parameters with the identical FOV 260 × 260 mm^2^:

1. A 3D $T_{1}$‐weighted axial image was acquired for anatomical imaging using magnetization prepared rapid gradient echo (MP‐RAGE) sequence with the following parameters: TE = 1.67 ms, TR = 1900 ms, voxel size = 1.5 × 1.5 × 2.5 mm^3^, and a scan time of 2 min, 3 s.
2. A 3D $B_{1}$ map was obtained using the Siemens clinical brain protocol. For $B_{1}$ mapping, the voxel size was 2.9 × 2.9 × 2.5 mm^3^ with a 57 s acquisition.
3. A diffusion tensor imaging (DTI) scan was performed with TE = 77 ms, TR = 3200 ms, voxel size = 2.5 × 2.5 × 2.5 mm^3^, *b*‐value = 0 and 1000 s/mm^2^, 30 diffusion directions, and a scan time of 3 min, 58 s.
4. MPF and $T_{1D}$ are acquired in a single scan. Spin‐lock prepared images were acquired based on 2D turbo spin echo (TSE) sequence. The spin‐lock parameters were set as follow (according to the results of Simulation Study 1): $\Delta \omega^{d\left(1\right)}/2\pi$=5kHz, $\omega_{1}^{d\left(1\right)}/2\pi$=500Hz, $\frac{∆\omega^{d\left(1\right)}}{\omega_{1}^{d\left(1\right)}}=\frac{∆\omega^{d\left(2\right)}}{\omega_{1}^{d\left(2\right)}}=\frac{∆\omega^{s\left(1\right)}}{\omega_{1}^{s\left(1\right)}}$=10, $\Delta \omega^{d\left(1\right)}=\Delta \omega^{s\left(1\right)}$, $\frac{∆\omega^{d\left(1\right)}}{∆\omega^{d\left(2\right)}}=\frac{\omega_{1}^{d\left(1\right)}}{\omega_{1}^{d\left(2\right)}}=5$, TSL = 80 ms. Switch times, $T_{s}$, are 0.5 ms for dual‐frequency spin‐lock and 40 ms for single‐frequency spin‐lock. The voxel size was 1.5 × 1.5 × 5 mm^3^, the number of slices was 3, TE was 9.2 ms, and TR was 2200 ms. Magnetization reset with $T_{1}$ recovery time was 1800 ms for fast acquisition strategy [33]. The acquisition time was 1 min 2 s for 3 spin‐lock prepared images per slice.

In addition, one volunteer underwent Z‐spectroscopic data acquisition using an MT‐weighted spoiled gradient echo (GRE) sequence with a Gaussian pulse for off‐resonance saturation with 11 Δ values (2, 3, 4, 6, 8, 10, 12, 16, 20, 32, and 36 kHz) and an independent $R_{1}$ map acquisition to calculate the MT parameters [27, 31].

Notably, the MPF and $T_{1D}$ acquisitions in phantom study were performed using the same parameters as in (4), except a FOV of 240 mm × 240 mm, a voxel size of 2 × 2 × 5 mm^3^.

#### 3.4.3 Data Processing and Analysis

To convert ${\text{RATIO}}_{\text{dosl}}$ to $T_{1D}$, we assumed that the MT model parameters (MPF, $R_{1b}$, $T_{2b}$, and R) remained constant. For agar phantoms, we used MPF = 2%, $R_{1b}=1\;\mathrm{Hz}$, $T_{2b}=10\;\mathrm{\mu s}$ and $R=210s^{-1}$ [36]. For PL161 phantoms, we used MPF = 15%, $R_{1b}=5\;\mathrm{Hz}$, $T_{2b}=17\;\mathrm{\mu s}$, and $R=46s^{-1}$, respectively [19, 37]. For healthy volunteers, we used the qMRLab (https://qmrlab.org/) to fit the Z‐spectroscopic data from one volunteer and estimated MPF = 13.6%. $R=20s^{-1},T_{2b}=9.7\mathrm{\mu s}$, while $R_{1b}$ was fixed at 2.9Hz based on literature [35].

${\text{RATIO}}_{\text{dosl}}$ maps were calculated from three spin‐lock prepared images $M_{d}^{\left(1\right)}$, $M_{d}^{\left(2\right)}$, and $M_{s}^{\left(1\right)}$ via Equation 11. Prior to this calculation, all spin‐lock prepared images were smoothed using a mean filter with a kernel size of 4 to reduce noise. The extreme values of ${\text{RATIO}}_{\text{dosl}}$ (e.g., > 5) have been masked. In the in vivo study, $T_{1D}$ maps were then obtained from ${\text{RATIO}}_{\text{dosl}}$ using both analytical estimation and dictionary matching. The dictionary was generated over a $T_{1D}$ range of 0–30 ms (step size $1\times 10^{-5}$ ms) and a $B_{1}$ range of 0.7–1.3 (step size $1\times 10^{-4}$). In the phantom studies, only dictionary matching was applied, as the current spin‐lock parameters were designed for white matter, which is suboptimal to be used for analytical estimation in phantoms.

$R_{\text{mpfsl}}$ maps were derived from $M_{d}^{\left(1\right)}$ and $M_{d}^{\left(2\right)}$, and converted to MPF maps using the fast MPF‐SL method with a dictionary approach [23, 28]. The in‐plane resolution of the $B_{1}$ map and DTI data were resampled to 1.5 × 1.5 mm^2^ using linear interpolation to match the $T_{1D}$ and MPF data.

To analyze $T_{1D}$ maps in ROIs of white matter, the $T_{1}$‐weighted images and DTI data were used for fiber bundles segmentation. The TractSeg opensource tool (https://github.com/MIC‐DKFZ/TractSeg) was employed to segment the fiber bundles of white matter [38]. In this study, the acquired slices for MPF measurement primarily included 16 regions of white matter fiber bundles: arcuate fascicle (AF_left, AF_right), anterior thalamic radiation (ATR_left, ATR_right), corpus callosum genu (CC_2), corpus callosum rostral body (CC_3), Corpus Callosum Posterior midbody (CC_5), Corpus callosum splenium (CC_7), cingulum (CG_left, CG_right), optic radiation (OR_left, OR_right), middle longitudinal fascicle (MLF_left, MLF_right), and fronto‐pontine tract (FPT_left, FPT_right). The mean and standard deviation of $T_{1D}$, as well as MPF and ${\text{RATIO}}_{\text{dosl}}$, were calculated for each white matter bundles. Additionally, the assessment of test–retest reproducibility of the in vivo study was performed, as described in Supporting Information 1.

## 4 Results

### 4.1 Simulation Studies

**FIGURE 3:**
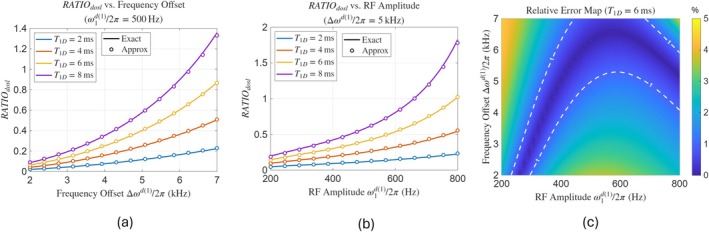
Comparison between the approximated analytical ${\text{RATIO}}_{\text{dosl}}$ and its exact numerical solution. (a) The relationship between ${\text{RATIO}}_{\text{dosl}}$ and $\Delta \omega^{d\left(1\right)}/2\pi$ (2–7kHz) at a fixed $\omega_{1}^{d\left(1\right)}/2\pi$ of 500 Hz. For each $T_{1D}$ (2, 4, 6, and 8 ms), the approximate results (markers) closely match the numerical solution curves (solid lines). (b) The relationship between ${\text{RATIO}}_{\text{dosl}}$ and $\omega_{1}^{d\left(1\right)}/2\pi$ (200–800 Hz) at a fixed $\Delta \omega^{d\left(1\right)}/2\pi$ of 5 kHz. The same agreement between approximate (marker) and exact numerical (solid lines) results is observed. (c) The relative error across the $\omega_{1}^{d\left(1\right)}/2\pi$ and $\Delta \omega^{d\left(1\right)}/2\pi$ range.

Figure 3 compares the approximated analytical ${\text{RATIO}}_{\text{dosl}}$ with its exact numerical solution. As shown in Figure 3a,b, the approximation (markers) demonstrates excellent agreement with the exact solution (solid lines). Under appropriate spin‐lock pulse parameters, the relative error remains below 1% (Figure 3c). These results confirm that the proposed approximation provides a reliable estimate, supporting its utility in practical applications.

**FIGURE 4:**
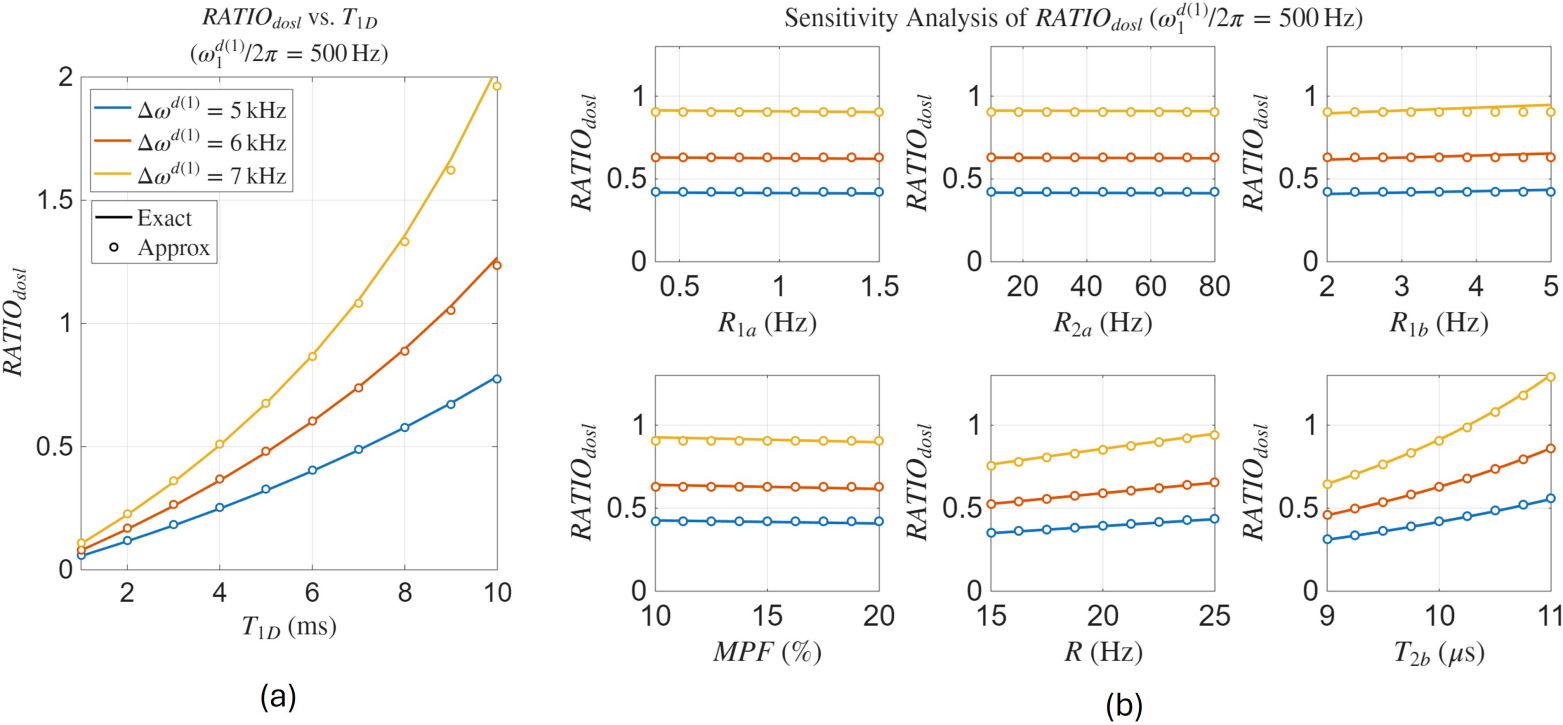
(a) The relationship between ${\text{RATIO}}_{\text{dosl}}$ and $T_{1D}$ at fixed $\omega_{1}^{d\left(1\right)}/2\pi$= 500Hz with selected $\Delta \omega^{d\left(1\right)}/2\pi$ = 5,6, and 7 kHz. Approximate results (markers) closely match numerical solutions (solid lines) over $T_{1D}$= 1–10 ms. For all $\Delta \omega^{d\left(1\right)}$ values, ${\text{RATIO}}_{\text{dosl}}$ increases with $T_{1D}$, confirming the high sensitivity of ${\text{RATIO}}_{\text{dosl}}$ to $T_{1D}$. (b) The relationship between ${\text{RATIO}}_{\text{dosl}}$ and other tissue parameters, including $R_{1a}$, $R_{2a}$, $R_{1b}$, MPF, R, and $T_{2b}$.

Figure 4a illustrates the sensitivity of ${\text{RATIO}}_{\text{dosl}}$ to $T_{1D}$. With $\omega_{1}^{d\left(1\right)}/2\pi$ fixed at 500 Hz and $\Delta \omega^{d\left(1\right)}/2\pi$ set to 5, 6, and 7 kHz, ${\text{RATIO}}_{\text{dosl}}$ increases markedly as $T_{1D}$ rises from 1 to 10 ms, demonstrating high sensitivity. Figure 4b depicts the sensitivity of ${\text{RATIO}}_{\text{dosl}}$ to other tissue parameters including $R_{1a}$, $R_{2a}$, $R_{1b}$, MPF, R, and $T_{2b}$. ${\text{RATIO}}_{\text{dosl}}$ is essentially independent of $R_{1a}$, $R_{2a}$, $R_{1b}$, and MPF. While it exhibits low sensitivity to the exchange rate R and pronounced sensitivity to $T_{2b}$, these parameters are typically treated as constants in human studies.

**FIGURE 5:**
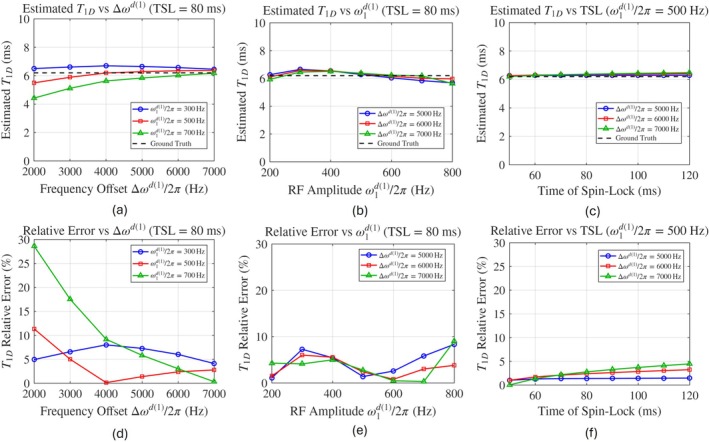
Simulation of the accuracy of $T_{1D}$ estimation as a function of spin‐lock pulse parameters. (a) Estimated $T_{1D}$ versus resonance frequency offset ($\Delta \omega^{d\left(1\right)}/2\pi$, 2–7 kHz) at fixed TSL = 80 ms and selected spin‐lock field strength $\omega_{1}^{d\left(1\right)}/2\pi$ values of 300, 500, and 700 Hz. (b) Estimated $T_{1D}$ versus spin‐lock field strength ($\omega_{1}^{d\left(1\right)}/2\pi$, 200–800 Hz) at fixed TSL = 80 ms and selected $\Delta \omega^{d\left(1\right)}/2\pi$ values of 5, 6, and 7 kHz. (c) Estimated $T_{1D}$ versus spin‐lock duration (TSL, 40–120 ms) at fixed $\omega_{1}^{d\left(1\right)}/2\pi$ = 500 Hz and selected ($\Delta \omega^{d\left(1\right)}/2\pi$ values of 5, 6, and 7 kHz. (d–f) Corresponding relative errors of the estimated $T_{1D}$ with respect to the ground‐truth value. The dashed line in (a–c) indicates the ground‐truth $T_{1D}=6.2$ ms.

Figure 5 presents an accuracy analysis of $T_{1D}$ estimation from measured ${\text{RATIO}}_{\text{dosl}}$ using analytical estimation method across various spin‐lock parameters ($\Delta \omega^{d\left(1\right)}$, $\omega_{1}^{d\left(1\right)}$, and TSL). Figure 5a,d suggest that $\Delta \omega^{d\left(1\right)}/2\pi$ values of 3–7 kHz combined with $\omega_{1}^{d\left(1\right)}/2\pi$ = 500 Hz are optimal, yielding relative errors below 5%. Figure 5b,e indicate that $\omega_{1}^{d\left(1\right)}/2\pi$ values of 500–700 Hz are favorable. The relationship between relative error and TSL varies by $\Delta \omega^{d\left(1\right)}$; notably, at $\Delta \omega^{d\left(1\right)}/2\pi$ = 5 kHz, the error is nearly independent of TSL. Based on these findings, we selected $\omega_{1}^{d\left(1\right)}/2\pi$ = 500 Hz, $\Delta \omega^{d\left(1\right)}/2\pi$ = 5 kHz, and TSL = 80 ms for in vivo experiments. These parameters fall within the scanner's SAR constraints and RF hardware limits.

**FIGURE 6:**
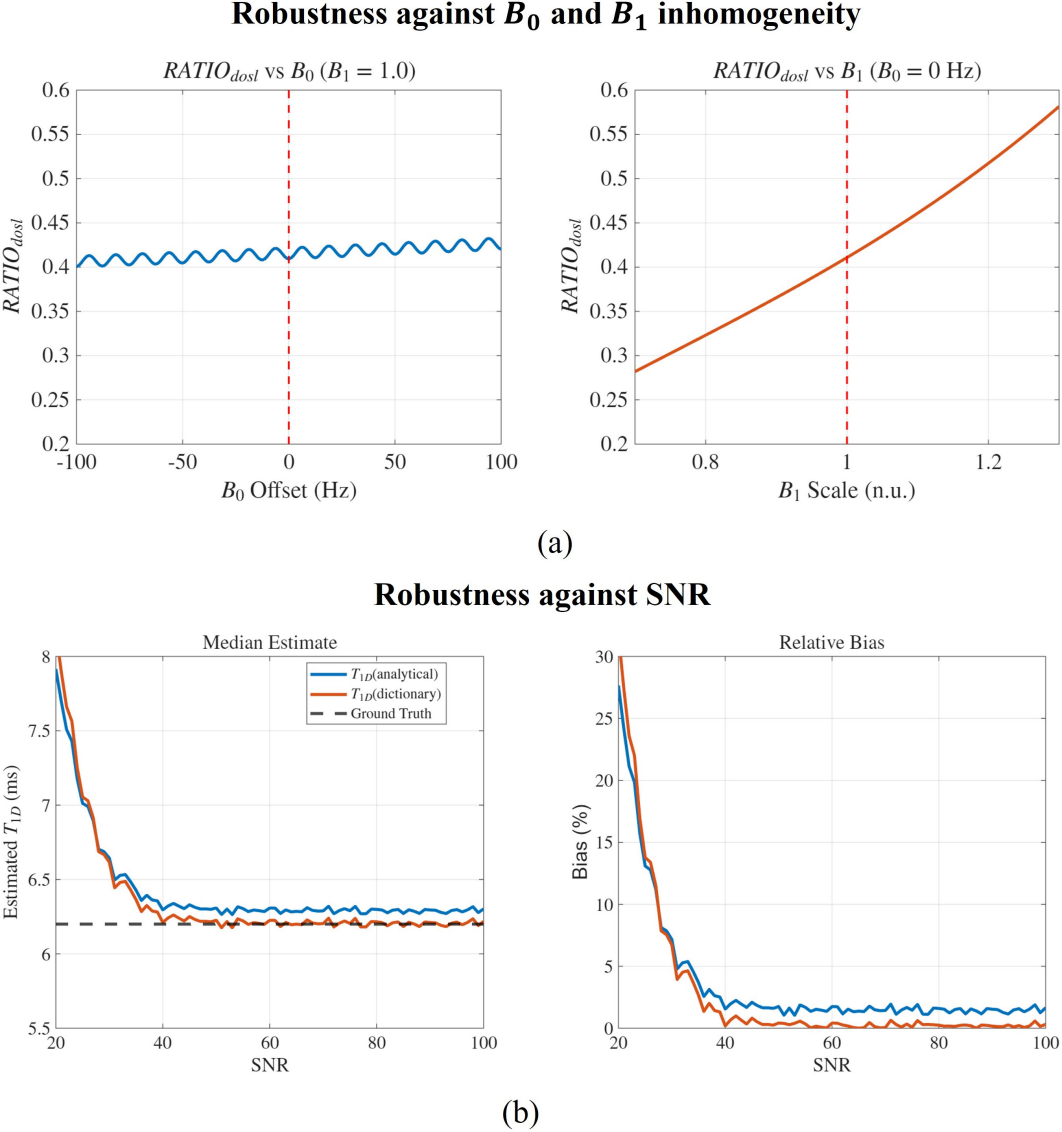
(a) Robustness of ${\text{RATIO}}_{\text{dosl}}$ to $B_{0}$ and $B_{1}$ inhomogeneity, displaying sensitivity to $B_{0}$ offsets (−100 to 100 Hz) and $B_{1}$ scaling factors (0.7 to 1.3 n.u.). (b) Robustness to SNR: a comparison of analytical estimation versus dictionary matching for $T_{1D}$. The plot shows the median estimate and relative bias compared to the ground‐truth $T_{1D}$= 6.2ms across an SNR range of 20–100.

Figure 6a displays the dependence of ${\text{RATIO}}_{\text{dosl}}$ on $B_{0}$ and $B_{1}$ inhomogeneity, alongside the relative error compared to the ground truth ($B_{0}$= 0 Hz, $B_{1}$= 1 n.u.). For $B_{0}$ between −100 and 100 Hz, ${\text{RATIO}}_{\text{dosl}}$ exhibits minimal variation and minor oscillations around the ground truth. However, consistent with theoretical predictions, ${\text{RATIO}}_{\text{dosl}}$ is sensitive to $B_{1}$ inhomogeneity because the lineshape depends on the actual RF amplitude. Notably, incorporating $B_{1}$ maps for retrospective correction of $T_{1D}$ effectively mitigates the impact of this inhomogeneity. As shown in Table 1A, the bias in $T_{1D}$ quantification due to field inhomogeneity is significantly reduced following this correction.

**TABLE 1:** Summary of robustness tests for $T_{1D}$ estimation.

A. Robustness against $B_{1}$ and $B_{0}$ inhomogeneity						
$B_{1}$(n.u.)	$B_{0}$ (Hz)	${\text{RATIO}}_{\text{dosl}}$	No $B_{1}$ correction		$B_{1}$ correction	
			$T_{1D}$(ms) (analytical estimation)	$T_{1D}$(ms) (dictionary matching)	$T_{1D}$(ms) (analytical estimation)	$T_{1D}$(ms) (dictionary matching)
0.7–1.3	−100 to 100	0.42 ± 0.09	6.52 ± 1.31	6.53 ± 1.56	6.38 ± 0.20	6.24 ± 0.26

B. Robustness against MT parameters (excluding $T_{2b}$)					
$R_{1b}$ (Hz)	MPF (%)	R (Hz)	${\text{RATIO}}_{\text{dosl}}$	$T_{1D}$(ms) (Analytical estimation)	$T_{1D}$(ms) (Dictionary matching)
2–5	10% to 20%	15–30	0.41 ± 0.02	6.29 ± 0.34	6.21 ± 0.40

*Note:* The ground truth $T_{1D}=6.2\;\mathrm{ms}$; the results are presented as mean ± standard deviation.

Table 1B details the results under variations of MT parameters. The mean ± standard deviation of ${\text{RATIO}}_{\text{dosl}}$, $T_{1D}$ (analytical estimation), and $T_{1D}$ (dictionary matching) were 0.41±0.02, 6.29±0.34, and 6.21±0.40 ms, respectively. Estimated $T_{1D}$ values show minimal bias (< 0.1 ms) relative to the ground truth (6.2 ms). These findings indicate that, assuming fixed MT parameters (excluding $T_{2b}$), the estimation of $T_{1D}$ is insensitive to variations in MT parameters.

Figure 6b presents the robustness against SNR. We calculated the median due to the presence of outliers at low SNR and relative bias for the estimated $T_{1D}$. This analysis confirms that SNR is critical for reliability, indicating that analytical estimation and dictionary matching methods require an SNR of at least ~40 to maintain a relative bias below 3%.

### 4.2 Phantom and In Vivo Studies

**FIGURE 7:**
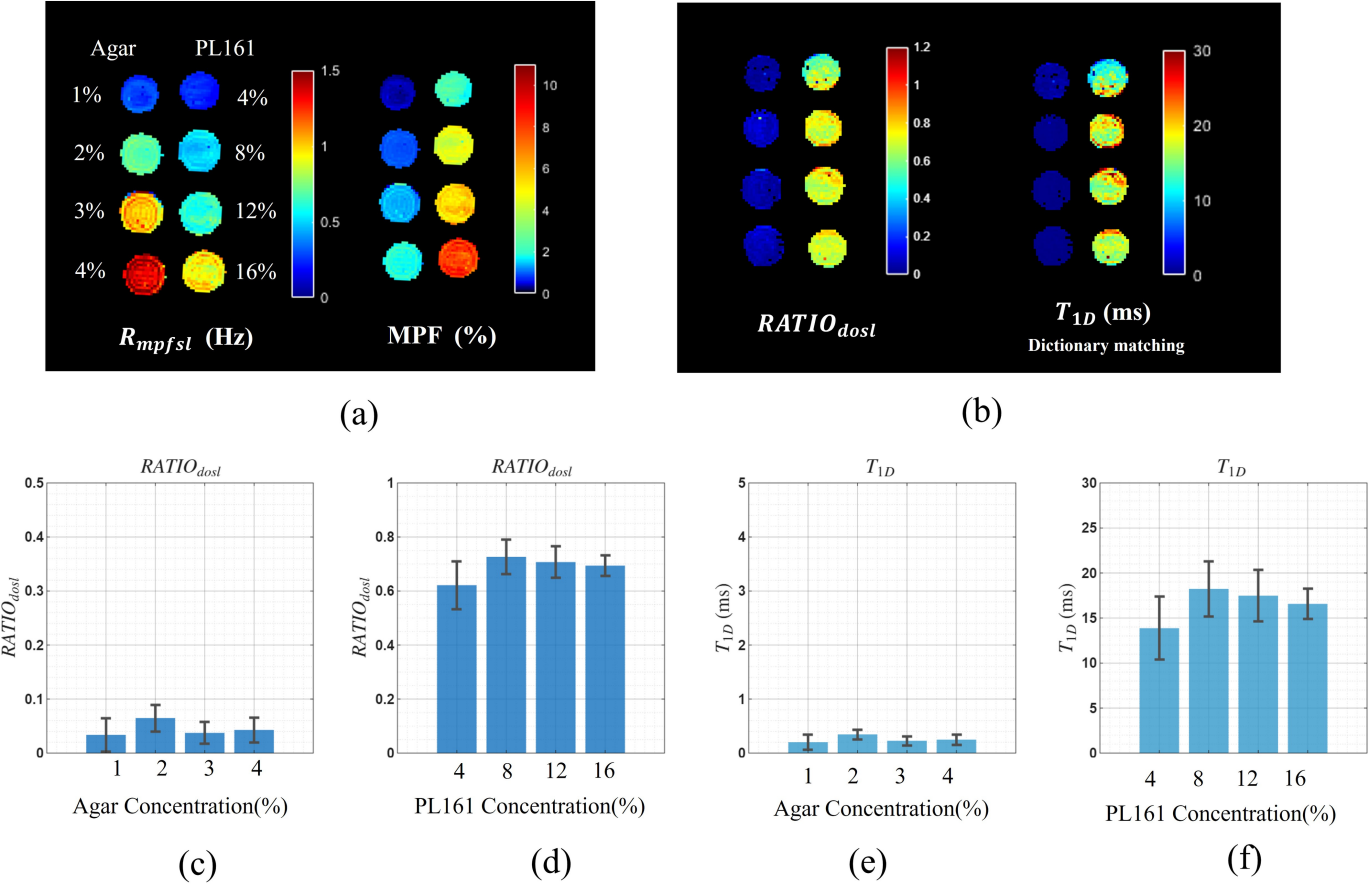
Results of phantom studies. (a) $R_{\text{mpfsl}}$ and MPF maps. (b) ${\text{RATIO}}_{\text{dosl}}$ and $T_{1D}$ map. The first column displays agar phantoms with concentrations of 1%, 2%, 3%, and 4% (from top to bottom); the second column displays PL161 phantoms with concentrations of 4%, 8%, 12%, and 16% (from top to bottom). (c)–(d) Bar plots of ${\text{RATIO}}_{\text{dosl}}$ for the agar and PL161 phantoms, respectively. Bars represent the mean within each phantom ROI, and error bars represent the standard deviation within ROI. (e)–(f) Corresponding bar plots of $T_{1D}$.

Figure 7a shows the MPF‐SL acquisitions for the agar and PL161 phantoms. The $R_{\text{mpfsl}}$ and the derived MPF are strongly associated with phantom concentration. Figure 7b presents the corresponding ${\text{RATIO}}_{\text{dosl}}$ and $T_{1D}$ maps. The ${\text{RATIO}}_{\text{dosl}}$ map highlights the contrast of the PL161 phantom, demonstrating its sensitivity to the ihMT effect. In the $T_{1D}$ maps, the long $T_{1D}$ of the PL161 phantom is confirmed by our method, whereas the agar phantom exhibits a notable MPF but negligible $T_{1D}$, consistent with its lack of dipolar behavior. Figure 7c–f show the relationships of ${\text{RATIO}}_{\text{dosl}}$ and $T_{1D}$ with phantom concentration. Both ${\text{RATIO}}_{\text{dosl}}$ and $T_{1D}$ show no obvious dependence on PL161 concentration overall.

**FIGURE 8:**
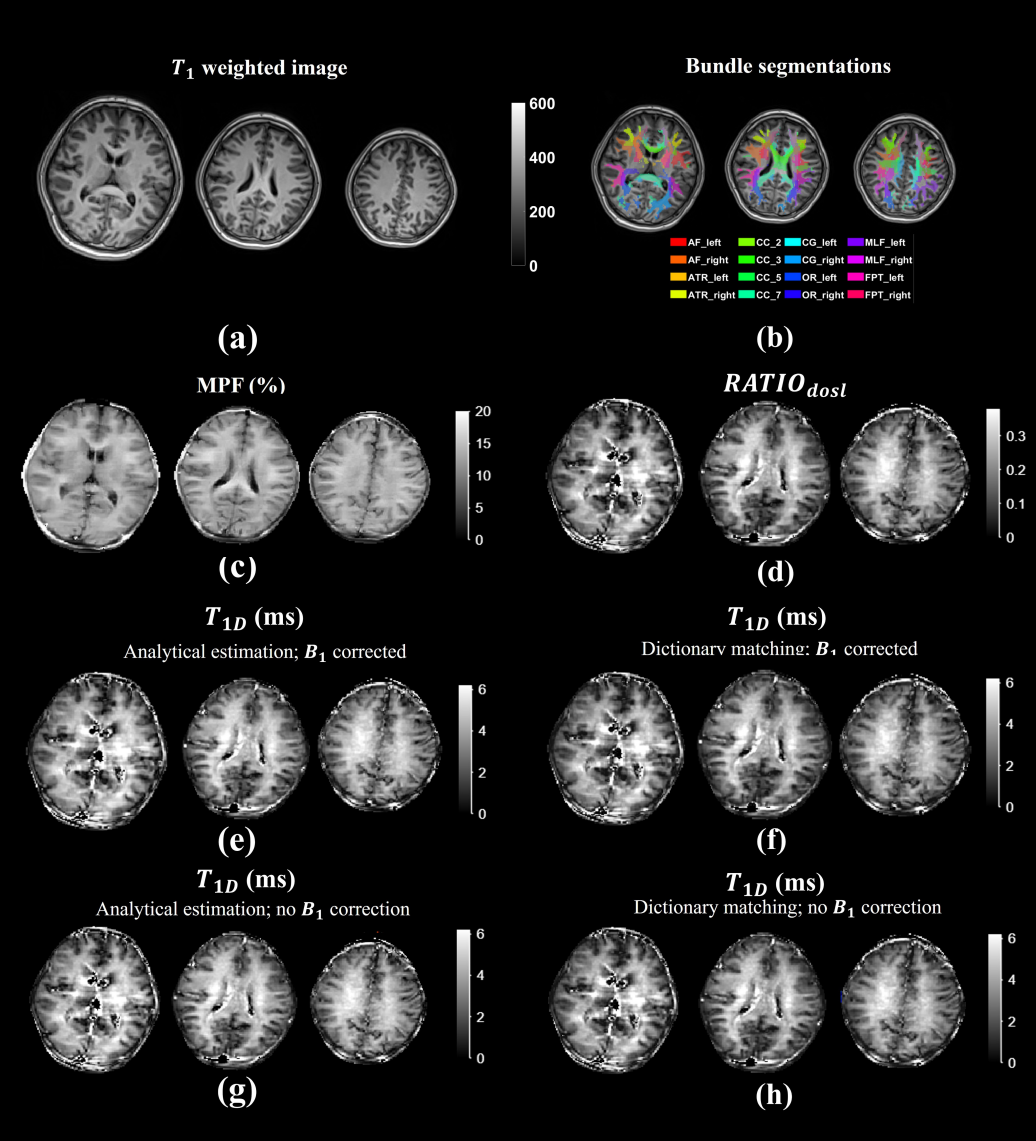
Representative results from one volunteer. (a) $T_{1}$‐weighted image of the acquired slices. (b) Bundle segmentation showing 16 major white matter fiber bundles. (c) MPF maps derived from MPF‐SL. (d) ${\text{RATIO}}_{\text{dosl}}$ maps. (e)–(f) $T_{1D}$ maps obtained via analytical estimation and dictionary matching, respectively, with $B_{1}$ correction. (g)–(h) Corresponding $T_{1D}$ maps obtained via analytical estimation and dictionary matching without $B_{1}$ correction.

Figure 8 presents in vivo results from one volunteer (V1). The $T_{1}$‐weighted anatomical image for the acquired slices and the 16 major white matter bundles are shown in Figure 8a,b. Figure 8c,d show the MPF maps and ${\text{RATIO}}_{\text{dosl}}$ maps, which exhibit different contrasts for white matter and indicate that these two parameters may carry different molecular signatures of tissues. $T_{1D}$ maps were derived from the ${\text{RATIO}}_{\text{dosl}}$ maps using analytical estimation and dictionary matching, with and without $B_{1}$ correction. The resulting $T_{1D}$ maps preserve a contrast similar to that of the ${\text{RATIO}}_{\text{dosl}}$ maps (Figure 8e–h). Results for the other volunteers are provided in Supporting Information 2. Table 2 summarizes the mean and standard deviation of MPF, ${\text{RATIO}}_{\text{dosl}}$, and the corresponding $T_{1D}$ across the 16 major white matter fiber bundles in 10 volunteers.

**TABLE 2:** Mean and standard deviation of MPF, ${\text{RATIO}}_{\text{dosl}}$, and $T_{1D}$ across the 16 major white matter fiber bundles.

	MPF (%)	${\text{RATIO}}_{\text{dosl}}$	$B_{1}$ correction		No $B_{1}$ correction	
			$T_{1D}$(ms) (analytical estimation)	$T_{1D}$(ms) (dictionary matching)	$T_{1D}$(ms) (analytical estimation)	$T_{1D}$(ms) (dictionary matching)
AF_left	14.51 ± 0.43	0.26 ± 0.01	4.46 ± 0.17	4.17 ± 0.19	4.10 ± 0.16	3.86 ± 0.17
AF_right	14.53 ± 0.38	0.27 ± 0.01	4.55 ± 0.19	4.27 ± 0.21	4.23 ± 0.17	3.99 ± 0.19
ATR_left	13.60 ± 0.56	0.24 ± 0.02	4.10 ± 0.30	3.81 ± 0.31	3.78 ± 0.28	3.53 ± 0.28
ATR_right	13.69 ± 0.36	0.24 ± 0.02	4.00 ± 0.29	3.69 ± 0.28	3.74 ± 0.26	3.47 ± 0.26
CC_2	13.93 ± 0.42	0.26 ± 0.01	4.34 ± 0.17	4.07 ± 0.20	4.04 ± 0.16	3.79 ± 0.18
CC_3	13.98 ± 0.49	0.28 ± 0.02	4.53 ± 0.25	4.42 ± 0.32	4.30 ± 0.27	4.08 ± 0.29
CC_5	14.04 ± 0.59	0.28 ± 0.02	4.74 ± 0.29	4.52 ± 0.34	4.36 ± 0.25	4.15 ± 0.28
CC_7	13.66 ± 0.43	0.26 ± 0.01	4.44 ± 0.19	4.14 ± 0.21	4.03 ± 0.17	3.77 ± 0.19
CG_left	14.18 ± 0.38	0.27 ± 0.01	4.59 ± 0.18	4.30 ± 0.20	4.21 ± 0.16	3.97 ± 0.18
CG_right	13.84 ± 0.33	0.24 ± 0.01	4.11 ± 0.15	3.79 ± 0.16	3.79 ± 0.14	3.52 ± 0.15
OR_left	13.47 ± 0.49	0.24 ± 0.03	4.25 ± 0.44	3.94 ± 0.43	3.84 ± 0.40	3.59 ± 0.39
OR_right	13.52 ± 0.35	0.25 ± 0.01	4.38 ± 0.19	4.07 ± 0.20	3.98 ± 0.18	3.73 ± 0.19
MLF_left	14.14 ± 0.45	0.27 ± 0.01	4.56 ± 0.17	4.26 ± 0.19	4.16 ± 0.16	3.91 ± 0.17
MLF_right	14.11 ± 0.43	0.29 ± 0.01	4.82 ± 0.18	4.59 ± 0.22	4.44 ± 0.18	4.22 ± 0.20
FPT_left	14.37 ± 0.40	0.30 ± 0.01	5.02 ± 0.22	4.80 ± 0.26	4.64 ± 0.20	4.45 ± 0.23
FPT_right	14.29 ± 0.35	0.28 ± 0.01	4.65 ± 0.20	4.40 ± 0.23	4.33 ± 0.18	4.11 ± 0.20

## 5 Discussion

Our proposed framework allows simultaneous quantification of $T_{1D}$ and MPF within a single scan. While MPF primarily reflects macromolecular content, $T_{1D}$ provides complementary sensitivity to microstructural integrity through the ihMT effect. Central to this approach is $\text{RATI}O_{\text{dosl}}$, a $T_{1D}$‐sensitive measure derived from the distinct relaxation rate $R_{\text{dosl}}$. Estimation of $\text{RATI}O_{\text{dosl}}$, and its subsequent conversion to $T_{1D}$ with $B_{1}$ correction, can be achieved using only three spin‐lock‐prepared images. Notably, two of these images can also be used for MPF quantification. Consequently, this simultaneous mapping strategy enables characterization of both tissue microstructure and macromolecular content within a single rapid scan. Recently, Hertanu et al. compared ihMTR and MPF with diffusion‐based modeling to investigate the microstructural correlates of white and gray matter [39]. Their findings highlight the potential of these parameters to disentangle distinct tissue mechanisms, supporting the premise that combined assessment of MPF and $T_{1D}$ may enhance tissue characterization. Accordingly, the proposed method may facilitate further clinical investigation of these parameters across a range of pathologies, both in the brain and in other organ systems.

In previous studies, Varma et al. reported an in vivo white matter $T_{1D}$ value of approximately 6.2 ms using a multi‐ihMTR approach [6] and approximately 3 ms under the assumption of a single MT compartment with a single $T_{1D}$ [11], whereas West et al. reported white matter $T_{1D}$ values of approximately 3.5–5.5 ms using an MRF‐based technique [20]. In the present study, we obtained white matter $T_{1D}$ values in the range of approximately 3.70–4.80 ms. Although these values are broadly consistent with prior reports, precise validation of the true $T_{1D}$ remains challenging. Moreover, the sensitivity of our method to $T_{2b}$ may introduce potential bias in the estimated $T_{1D}$, which could also contribute to differences relative to prior reports. To provide indirect support for our $T_{1D}$ estimates, we performed in vivo experiments in which the $T_{s}$ of the dual frequency spin‐lock preparation was varied from 0.5 to 20 ms. The results showed that white matter was strongly highlighted when the dual‐frequency spin‐lock $T_{s}$ was ≤ 4 ms (Figure S1.2 in Supporting Information 1), which provides indirect support for the obtained $T_{1D}$ values based on the expected $T_{1D}$‐filtering effect.

For the proposed method, because ${\text{RATIO}}_{\text{dosl}}$ is derived from different $R_{1\rho }$ measurements under a constant spin‐lock field direction, contributions from the water pool are effectively cancelled. Simulation studies further indicate that ${\text{RATIO}}_{\text{dosl}}$ exhibits only weak dependence on other MT parameters (e.g., MPF, R, and R_1b_).

Prolonged scan times often limit the clinical utility of $T_{1D}$ assessment [6, 20]. Our approach addresses this by requiring only three spin‐lock prepared images for joint $T_{1D}$ and MPF quantification. Although this study demonstrated the technique in 2D slices, extending the method to whole‐brain coverage is feasible using fast 3D MRI acquisition strategies [40, 41, 42]. In previous work [42], using four spin‐lock prepared images combined with 3D FSE/TSE acquisition, whole‐brain coverage of $T_{1\rho }$ quantification could be achieved within 5 min. Consequently, by leveraging strategies that rely solely on three spin‐lock prepared images, we estimate the proposed method can achieve whole‐brain coverage in 5 min.

Our method employs the least negative eigenvalue to model signal evolution, an approach traditionally assumed to necessitate long saturation times [29]. However, our analysis indicates that this approximation remains robust at short spin‐lock durations (e.g., 80 ms) under specific experimental conditions. By utilizing large frequency offsets relative to the spin‐lock amplitude $\left(∆\omega \gg \omega_{1}\right)$, the system enters a regime where magnetization decay is dominated by a single component. In this context, contributions from faster‐decaying modes are negligible, allowing the least negative eigenvalue to characterize signal evolution as a mono‐exponential process. Moreover, at the large frequency offset used in this study ($\Delta \omega^{d\left(1\right)}/2\pi$=5kHz), CEST and NOE contributions are expected to be negligible [23]. In addition, three‐pool simulations that explicitly incorporated a CEST pool showed negligible effects on ${\text{RATIO}}_{\text{dosl}}$ across broad CEST parameter ranges (Figure S1.3 in Supporting Information 1).

In MT imaging, saturation efficiency is strongly influenced by the time‐averaged RF power, which is proportional to the time‐averaged $B_{1}^{2}$. Under the same peak‐power and SAR constraints, a piecewise‐constant RF waveform may provide higher effective average saturation than a conventional Gaussian RF pulse. Moreover, piecewise‐constant RF irradiation is more readily incorporated into the Provotorov formalism. In conventional saturation‐based methods, however, the use of square‐wave RF irradiation is associated with a trade‐off involving direct water saturation, partly because of its broader spectral characteristics. By contrast, in the proposed spin‐lock‐based framework, square‐wave RF pulses can be incorporated into the preparation process in a manner that is less constrained by this trade‐off.

In this study, we compared analytical estimation and dictionary matching for $T_{1D}$ quantification. At SNR ≥40, both approaches yielded low error; analytical estimation showed a small residual bias due to model approximations, whereas dictionary matching achieved slightly higher accuracy. Analytical estimation offers fast computation without the memory and runtime overhead associated with large dictionaries; however, its accuracy can depend on careful optimization of acquisition parameters to control bias across tissue types. Dictionary matching is, in principle, applicable to arbitrary acquisition settings. Overall, the choice of $T_{1D}$ estimation approach should be guided by the study's SNR conditions and acquisition design.

Beyond quantitative $T_{1D}$ mapping, the proposed framework may also be adapted for $T_{1D}$‐weighted contrast generation. Because the contrast mechanism depends on the relationship between the switching time $T_{s}$ and the underlying $T_{1D}$, adjustment of $T_{s}$ may enable preferential sensitivity to components with short, intermediate, or long $T_{1D}$ values. In such cases, simple approximate metrics derived from a limited number of images, for example $\left(M_{s}^{\left(1\right)}-M_{d}^{\left(1\right)}\right)/M_{d}^{\left(1\right)}$, may offer a practical alternative to explicit $T_{1D}$ quantification. This strategy may be useful in applications where the primary goal is to enhance $T_{1D}$‐dependent contrast rather than to obtain absolute parametric estimates. Further work is needed to optimize and validate such acquisition schemes for selective component weighting.

Looking ahead, other spin‐lock‐based quantitative MT techniques, such as pulsed spin‐lock approaches [25], may also be leveraged for dipolar‐order quantification. Pulsed spin‐lock methods can mitigate RF hardware limitations, offering particular benefits for body imaging and low‐field applications where power constraints are pronounced.

Although our theoretical analysis and experimental results support the reliability and clinical feasibility of spin‐lock–based $T_{1D}$ quantification, several areas warrant further investigation to refine the technique:

**The influence of** $T_{2b}$ **on** $T_{1D}$ **quantification**: The lineshape parameter $T_{2b}$ is often assumed to be constant in the quantification of both MT and ihMT parameters [20, 33], and we adopted the same assumption in the present study. However, our simulations indicate that ${\text{RATIO}}_{\text{dosl}}$ is sensitive to $T_{2b}$, suggesting that variations in this parameter may introduce bias into the estimated $T_{1D}$. To further assess this effect, we performed additional simulations to evaluate the sensitivity of the estimated $T_{1D}$ to $T_{2b}$ (Figure S1.4 in Supporting Information 1). Reported $T_{2b}$ values in brain tissue range from 9.2 to 12.3 μs [28, 43, 44], with minimal differences between white and gray matter and a standard deviation of 1.5–2 μs [31, 43]. In addition, $T_{2b}$ has been reported to increase by approximately 1 μs in multiple sclerosis lesions [45]. These findings suggest that future work should consider incorporating $T_{2b}$ estimation into the existing framework for joint $T_{1D}$ and MPF mapping. Furthermore, mismatch between the assumed super‐Lorentzian lineshape and the true bound‐pool lineshape may also bias $T_{1D}$ quantification and warrants further investigation.
**Modeling assumptions for the** $T_{1D}$ **component:** Our current model assumes a single $T_{1D}$ component and a dipolar‐order fraction of unity. However, previous studies have suggested that the ihMT effect may be influenced by multiple dipolar components and by dipolar fractions that deviate from unity [46, 47]. Under such conditions, the $T_{1D}$ estimated with the present framework should be interpreted as an effective parameter rather than as a direct measure of a single underlying physical component. Consequently, model mismatch may introduce bias into $T_{1D}$ quantification, and the magnitude of this bias may depend on the relative contributions of different components as well as on the acquisition settings. The current framework therefore represents a first‐order approximation. Future work should investigate more comprehensive models to improve quantification accuracy and to better characterize the underlying physical mechanisms.
**Validation and biological interpretation:** The potential orientation dependence of this specific spin‐lock approach remains to be established [48], although prior studies have suggested that spin‐lock techniques may be relatively orientation‐insensitive [26, 27]. More broadly, the value of the proposed method will depend on validation against established reference approaches. The development of standardized benchmarks will therefore be important for consistent evaluation across studies. Future work should compare the proposed framework with existing $T_{1D}$ quantification techniques and further establish its biological and clinical relevance through correlations with histology and pathological processes, such as demyelination. Such validation will be essential to define the accuracy, specificity, and translational potential of the method, and to clarify its role in routine research and clinical applications.

## 6 Conclusions

We demonstrated a novel and rapid off‐resonance spin‐lock technique for quantifying the dipolar relaxation time $T_{1D}$. The ability to measure both MPF and $T_{1D}$ within a single scan highlights the potential clinical utility of this approach and provides a promising pathway toward integrating molecular microstructural imaging into clinical practice.

## Author Contributions

**Zijian Gao:** conceptualization, data curation, software, investigation, methodology, project administration, writing – original draft, visualization. **Qianxue Shan:** investigation, methodology, formal analysis, validation. **Ziqin Zhou:** data curation, resources, software. **Ziqiang Yu:** data curation, software, methodology. **Weitian Chen:** conceptualization, investigation, project administration, supervision, methodology, funding acquisition, writing – review and editing.

## Conflicts of Interest

Weitian Chen is a shareholder of Illuminatio Medical Technology Limited.

## Supporting information




**Figure S1:1.** Bland–Altman plots and correlation plots for the human studies across the 16 major white matter fiber bundles in 10 volunteers.
**Figure S1:2.** ${\text{RATIO}}_{\text{dosl}}$ maps acquired with dual‐frequency spin‐lock using $T_{s}$ = 0.5, 1, 2, 4, 10, and 20 ms, while $T_{s}$ of the single‐frequency spin‐lock was fixed at 40 ms.
**Figure S1:3**. Sensitivity of $\text{RATI}O_{\text{dosl}}$ to CEST‐related parameters in a three‐pool simulation.
$\text{RATI}O_{\text{dosl}}$ is shown as a function of $R_{2}^{\text{CEST}}$, $k_{\text{CEST}}$, and CEST pool size ratio $f_{\text{CEST}}$, with curves corresponding to $\Delta \omega^{d\left(1\right)}/2\pi =5,6,7$ kHz. Baseline parameters were $R_{2}^{\text{CEST}}=67$ Hz, $\Delta \omega_{\text{CEST}}=1.9$ ppm, $k_{\text{CEST}}=1500\;s^{-1}$, and $f_{\text{CEST}}=0.14\%$. Parameter ranges were $R_{2}^{\text{CEST}}=30–75$ Hz, $k_{\text{CEST}}=1000–2500\;s^{-1}$, and $f_{\text{CEST}}=0.1\%–1\%$. The spin‐lock amplitude was fixed at $\omega_{1}^{d\left(1\right)}/2\pi =500$ Hz (FSL).
**Figure S1:4.** The sensitivity of estimated $T_{D}$ against $T_{2b}$ ranged from 9 to 11 μs.

**Figure S2:1.** MPF map, ${\text{RATIO}}_{\text{dosl}}$ map, and $T_{1D}$ maps (derived using analytical estimation and dictionary matching) with $B_{1}$ correction in volunteer V1.
**Figure S2:2.** MPF map, ${\text{RATIO}}_{\text{dosl}}$ map, and $T_{1D}$ maps (derived using analytical estimation and dictionary matching) with $B_{1}$ correction in the V1 retest.
**Figure S2:3.** MPF map, ${\text{RATIO}}_{\text{dosl}}$ map, and $T_{1D}$ maps (derived using analytical estimation and dictionary matching) with $B_{1}$ correction in volunteer V2.
**Figure S2:4.** MPF map, ${\text{RATIO}}_{\text{dosl}}$ map, and $T_{1D}$ maps (derived using analytical estimation and dictionary matching) with $B_{1}$ correction in the V2 retest.
**Figure S2:5.** MPF map, ${\text{RATIO}}_{\text{dosl}}$ map, and $T_{1D}$ maps (derived using analytical estimation and dictionary matching) with $B_{1}$ correction in volunteer V3.
**Figure S2:6.** MPF map, ${\text{RATIO}}_{\text{dosl}}$ map, and $T_{1D}$ maps (derived using analytical estimation and dictionary matching) with $B_{1}$ correction in the V3 retest.
**Figure S2:7.** MPF map, ${\text{RATIO}}_{\text{dosl}}$ map, and $T_{1D}$ maps (derived using analytical estimation and dictionary matching) with $B_{1}$ correction in volunteer V4.
**Figure S2:8.** MPF map, ${\text{RATIO}}_{\text{dosl}}$ map, and $T_{1D}$ maps (derived using analytical estimation and dictionary matching) with $B_{1}$ correction in the V4 retest.
**Figure S2:9.** MPF map, ${\text{RATIO}}_{\text{dosl}}$ map, and $T_{1D}$ maps (derived using analytical estimation and dictionary matching) with $B_{1}$ correction in volunteer V5.
**Figure S2:10.** MPF map, ${\text{RATIO}}_{\text{dosl}}$ map, and $T_{1D}$ maps (derived using analytical estimation and dictionary matching) with $B_{1}$ correction in the V5 retest.
**Figure S2:11.** MPF map, ${\text{RATIO}}_{\text{dosl}}$ map, and $T_{1D}$ maps (derived using analytical estimation and dictionary matching) with $B_{1}$ correction in volunteer V6.
**Figure S2:12.** MPF map, ${\text{RATIO}}_{\text{dosl}}$ map, and $T_{1D}$ maps (derived using analytical estimation and dictionary matching) with $B_{1}$ correction in the V6 retest.
**Figure S2:13.** MPF map, ${\text{RATIO}}_{\text{dosl}}$ map, and $T_{1D}$ maps (derived using analytical estimation and dictionary matching) with $B_{1}$ correction in volunteer V7.
**Figure S2:14.** MPF map, ${\text{RATIO}}_{\text{dosl}}$ map, and $T_{1D}$ maps (derived using analytical estimation and dictionary matching) with $B_{1}$ correction in the V7 retest.
**Figure S2:15.** MPF map, ${\text{RATIO}}_{\text{dosl}}$ map, and $T_{1D}$ maps (derived using analytical estimation and dictionary matching) with $B_{1}$ correction in volunteer V8.
**Figure S2:16.** MPF map, ${\text{RATIO}}_{\text{dosl}}$ map, and $T_{1D}$ maps (derived using analytical estimation and dictionary matching) with $B_{1}$ correction in the V8 retest.
**Figure S2:17.** MPF map, ${\text{RATIO}}_{\text{dosl}}$ map, and $T_{1D}$ maps (derived using analytical estimation and dictionary matching) with $B_{1}$ correction in volunteer V9.
**Figure S2:18.** MPF map, ${\text{RATIO}}_{\text{dosl}}$ map, and $T_{1D}$ maps (derived using analytical estimation and dictionary matching) with $B_{1}$ correction in the V9 retest.
**Figure S2:19.** MPF map, ${\text{RATIO}}_{\text{dosl}}$ map, and $T_{1D}$ maps (derived using analytical estimation and dictionary matching) with $B_{1}$ correction in volunteer V10.
**Figure S2:20.** MPF map, ${\text{RATIO}}_{\text{dosl}}$ map, and $T_{1D}$ maps (derived using analytical estimation and dictionary matching) with $B_{1}$ correction in the V10 retest.

## Data Availability

The data that support the findings of this study are available on request from the corresponding author. The data are not publicly available due to privacy or ethical restrictions.
