# Supplementary material for: Dipolar Order Mapping Based on Spin‐Lock Magnetic Resonance Imaging
Source: NMR Biomed. 2026 Jun 17;39(7):e70331. doi: 10.1002/nbm.70331 (PMC13275185; doi:10.1002/nbm.70331)
Supplement: Supplementary file 1 — Figure S1:1. Bland–Altman plots and correlation plots for the human studies across the 16 major white matter fiber bundles in 10 volunteers. Figure S1:2. RATIOdosl maps acquired with dual‐frequency spin‐lock using Ts = 0.5, 1, 2, 4, 10, and 20 ms, while Ts of the single‐frequency spin‐lock was fixed at 40 ms. Figure S1:3. Sensitivity of RATIOdosl to CEST‐related parameters in a three‐pool simulation. RATIOdosl is shown as a function of R2CEST, kCEST, and CEST pool size ratio fCEST, with curves corresponding to Δωd1/2π=5,6,7 kHz. Baseline parameters were R2CEST=67 Hz, ΔωCEST=1.9 ppm, kCEST=1500s−1, and fCEST=0.14%. Parameter ranges were R2CEST=30–75 Hz, kCEST=1000–2500s−1, and fCEST=0.1%–1%. The spin‐lock amplitude was fixed at ω1d1/2π=500 Hz (FSL). Figure S1:4. The sensitivity of estimated TD against T2b ranged from 9 to 11 μs. [file NBM-39-e70331-s001.docx]

# **Supporting Information 1**

# **1. The derivation of** $\boldsymbol{R}_{\boldsymbol{dosl}}$ **based on Bloch–McConnell–Provotorov equation**

Trott and Palmer reported that $R_{1\rho}$ is associated with the least negative eigenvalue of the Bloch–McConnell matrix system^1^. Zaiss et al. extended this approach to the two-pool model comprising the water pool and the MT pool^2^. In this study, we apply the same framework to the Bloch–McConnell–Provotorov matrix system that incorporates dipolar order in the MT pool. Under the single-frequency spin-lock irradiation, the $R_{1\rho}^{single}$ with dipolar order can be divided into $R_{W}$ and $R_{MT}^{s}$. The effective water relaxation rate $R_{W}$ is given by:

$R_{W}=R_{1a}{cos}^{2}\varphi+R_{2a}{sin}^{2}\varphi$ (S.1)

where ${cos}^{2}\varphi=\frac{{\Delta\omega}^{2}}{\omega_{1}^{2}+{\Delta\omega}^{2}}$ and ${sin}^{2}\varphi=\frac{\omega_{1}^{2}}{\omega_{1}^{2}+{\Delta\omega}^{2}}$, and $\varphi$ represents the direction of the spin-lock field. $R_{MT}^{s}$ is the relaxation rate associated with MT pool under single-frequency spin-lock irradiation. To accurately approximate the effective $R_{MT}^{s}$, $A$ in Bloch-McConnel-Provotorov matrix system (Eq.3) can be shifted to $A^{'}=A-diag(R_{w})$ ^2^,which yields:

$A^{'}=\left( \begin{matrix} -r_{2a} & -\text{Δω} & 0 & 0 & 0 \\ +\text{Δω} & -r_{2a} & +\omega_{1} & 0 & 0 \\ 0 & -\omega_{1} & -r_{1a}-k_{ab} & k_{ba} & 0 \\ 0 & 0 & k_{ab} & -r_{1b}-k_{ba} & R_{rfb}\Delta\omega\\ 0 & 0 & 0 & R_{rfb}\frac{\Delta\omega}{D^{2}} & r_{d}-\frac{1}{T_{1D}} \end{matrix} \right)$ (S.2)

Where $r_{1a}=R_{1a}-R_{w}$, $r_{2a}=R_{2a}-R_{w}$, $r_{1b}=R_{1b}+R_{rfb}-R_{w}$, and $r_{d}=-R_{rfb}\left( \frac{\Delta\omega}{D} \right)^{2}-R_{w}$. Using Mathematica (Wolfram Research), the least negative eigenvalue of the shifted system $A^{'}$ as follows:

$R_{MT}^{s}=\frac{R_{rfb}^{2}T_{1D}\Delta\omega^{2}\cdot\mathcal{N}_{1}+D^{2}(1-r_{d}T_{1D})\cdot\mathcal{N}_{2}}{{-R}_{rfb}^{2}T_{1D}\Delta\omega^{2}\cdot\mathcal{G}_{1}+D^{2}\cdot\mathcal{G}_{2}}$ (S.3)

$\begin{aligned} \mathcal{N}_{1}&=-\left( k_{ab}+r_{1a} \right)\left( \frac{r_{2a}^{2}}{\Delta\omega^{2}}+1 \right)-r_{2a}\frac{\omega_{1}^{2}}{\Delta\omega^{2}} \end{aligned}$ (S.4)

$\begin{aligned} \mathcal{N}_{2}&=(\left( k_{ba}r_{1a}+\left( k_{ab}+r_{1a} \right)r_{1b} \right)\left( \frac{r_{2a}^{2}}{\Delta\omega^{2}}+1 \right) \\ & +\left( k_{ba}+r_{1b} \right)r_{2a}\frac{\omega_{1}^{2}}{\Delta\omega^{2}}) \end{aligned}$ (S.5)

$\begin{aligned} \mathcal{G}_{1}&=\frac{{2r}_{2a}\left( \left( k_{ab}+r_{1a} \right) \right)}{\Delta\omega^{2}}+\frac{r_{2a}^{2}}{\Delta\omega^{2}}+1+\frac{\omega_{1}^{2}}{\Delta\omega^{2}} \end{aligned}$ (S.6)

The term $\mathcal{G}_{2}$ can be further decomposed as:

$\mathcal{G}_{2}=(\mathcal{G}_{2a}+\mathcal{G}_{2b}+\mathcal{G}_{2c}+\mathcal{G}_{2d}+\mathcal{G}_{2e})$ (S.7)

Each component is defined as follows:

$\begin{aligned} \mathcal{G}_{2a}&=\frac{2r_{1b}r_{2a}}{\Delta\omega^{2}}r_{1a}+{(r}_{1a}+r_{1b}+r_{1a}r_{1b}T_{1D})\frac{r_{2a}^{2}}{\Delta\omega^{2}} \end{aligned}$ (S.8)

$\begin{aligned} \mathcal{G}_{2b}&=-\frac{2r_{1b}r_{2a}r_{d}T_{1D}}{\Delta\omega^{2}}r_{1a}-{(r}_{1a}r_{d}T_{1D}+r_{1b}r_{d}T_{1D})\frac{r_{2a}^{2}}{\Delta\omega^{2}} \end{aligned}$ (S.9)
$\begin{aligned} \mathcal{G}_{2c}&=\frac{k_{ba}r_{2a}\left( 2+r_{2a}T_{1D}-2r_{d}T_{1D} \right)}{\Delta\omega^{2}}r_{1a}+k_{ab}\left( 1-r_{d}T_{1D}+T_{1D} \right)\frac{r_{2a}^{2}}{\Delta\omega^{2}} \\ & +2r_{1b}\left( 1-r_{d}T_{1D} \right)\frac{k_{ab}r_{2a}}{\Delta\omega^{2}} \end{aligned}$ (S.10)

$\begin{aligned} \mathcal{G}_{2d}&=r_{1a}(1+k_{ba}T_{1D}+r_{1b}T_{1D}-r_{d}T_{1D}) \\ & +{(r}_{1b}+k_{ab}+k_{ba}\left( \frac{r_{2a}^{2}}{\Delta\omega^{2}}+1 \right))(1-r_{d}T_{1D}) \\ & +k_{ab}r_{1b}T_{1D} \end{aligned}$ (S.11)

$\begin{aligned} \mathcal{G}_{2e}&=\left( k_{ba}+r_{1b}+r_{2a}+\left( k_{ba}+r_{1b} \right)r_{2a}T_{1D} \right. \\ & \left. -\left( k_{ba}+r_{1b}+r_{2a} \right)r_{d}T_{1D} \right)\frac{\omega_{1}^{2}}{\Delta\omega^{2}} \\ & \end{aligned}$ (S.12)

Considering our spin-lock pulse implementation and tissue parameters of white matter, $\Delta\omega/\omega_{1}\gg1$ , $\Delta\omega\gg r_{2a}$, $\Delta\omega\gg k_{ab}$ , $R_{rfb}{\gg R_{1b}-R}_{1a}$, and $R_{rfb}\left( \frac{\Delta\omega}{D} \right)^{2}\gg R_{w}$ are satisfied. Subsequentially , we have $R_{W}\cong R_{1a}$, $r_{1a}\cong0$, $\omega_{1}^{2}/\Delta\omega^{2} \cong0$, $r_{2a}^{2}/\Delta\omega^{2}\cong0$, $k_{ab}r_{2a}/\Delta\omega^{2}\cong0$, $r_{1b}\cong R_{rfb}$ ,and $r_{d}\cong-R_{rfb}\left( \frac{\Delta\omega}{D} \right)^{2}$. Substituting these into Eq. S.3-12, we obtained $\begin{aligned} \mathcal{N}_{1}&\approx-k_{ab} \end{aligned}$, $\mathcal{N}_{2}\approx{k_{ab}r}_{1b}$, $\mathcal{G}_{1}\approx1$, and by ignoring $\mathcal{G}_{2a}$, $\mathcal{G}_{2b}$, $\mathcal{G}_{2c}$, and $\mathcal{G}_{2e}$. $\mathcal{G}_{2d}$ can be simplified to

$\mathcal{G}_{2d}= {(r}_{1b}+k_{ab}+k_{ba})(1+R_{rfb}\left( \frac{\Delta\omega}{D} \right)^{2}T_{1D})+k_{ab}r_{1b}T_{1D}$ (S.13)

Approximate $R_{MT}^{s}$ is further updated:

$$\begin{aligned} R_{MT}^{s}&=\frac{-R_{rfb}^{2}T_{1D}{(\frac{\Delta\omega}{D})}^{2}k_{ab}+(1+{R_{rfb}\left( \frac{\Delta\omega}{D} \right)}^{2}T_{1D}){k_{ab}r}_{1b}}{{-R}_{rfb}^{2}T_{1D}{(\frac{\Delta\omega}{D})}^{2}+{(r}_{1b}+k_{ab}+k_{ba})(1+R_{rfb}\left( \frac{\Delta\omega}{D} \right)^{2}T_{1D})+k_{ab}r_{1b}T_{1D}} \\ &=\frac{k_{ab}R_{rfb}}{\left( k_{ab}+k_{ba} \right)\left( 1+R_{rfb}\left( \frac{\Delta\omega}{D} \right)^{2}T_{1D} \right)+{R_{rfb}(k}_{ab}T_{1D}+1)} \\ & \end{aligned}$$

(S.14)

It is notable that ${k_{ab}T}_{1D}+1\cong1$ and $k_{ab}=f_{b}k_{ba}$. Therefore, the approximate $R_{MT}^{s}$ is further given with $k_{ab}=f_{b}k_{ba}$:

$R_{MT}^{s}\cong\frac{f_{b}k_{ba}R_{rfb}}{k_{ba}(f_{b}+1)\left( 1+R_{rfb}\left( \frac{\Delta\omega}{D} \right)^{2}T_{1D} \right)+R_{rfb}}$ (S.15)

Similarly, $R_{MT}^{d}$ ,the relaxation rate associated with MT pool without dipolar order under dual-frequency spin-lock conditions, from $R_{1\rho}^{dual}$ can be derived by:

$R_{MT}^{d}\cong\frac{f_{b}k_{ba}R_{rfb}}{k_{ba}(f_{b}+1)+R_{rfb}}$ (S.16)

When the single-frequency spin-lock with ${\Delta\omega}^{s}\text{and }{\omega_{1}}^{s}$ and dual-frequency spin-lock with ${\Delta\omega}^{d}\text{and }{\omega_{1}}^{d}$ are applied with the same direction of spin-lock field ($\frac{\Delta\omega^{d}}{\omega_{1}^{d}}=\frac{\Delta\omega^{s}}{\omega_{1}^{s}}$) , the water pool contribution $R_{W}$ can be remove by the difference between $R_{1\rho}^{single}$ and $R_{1\rho}^{dual}$ . Consequently, we defined a specific relaxation rate $R_{dosl}$ associated with the ihMT effect:

$\begin{aligned} R_{dosl}&=R_{1\rho}^{dual}-R_{1\rho}^{single}=R_{MT}^{d}-R_{MT}^{s} \\ &=f_{b}k_{ba}\left[ \frac{R_{rfb}^{d}}{k_{ba}(f_{b}+1)+R_{rfb}^{d}}-\frac{R_{rfb}^{s}}{k_{ba}(f_{b}+1)(R_{rfb}^{s}\left( \frac{\Delta\omega^{s}}{D} \right)^{2}T_{1D}+1)+R_{rfb}^{s}} \right] \end{aligned}$ (S.17)

$R_{rfb}^{s}$ and $R_{rfb}^{d}$ correspond to lineshape under single-frequency and dual-frequency spin-lock conditions, respectively

# **2. Test-retest reproducibility for *in vivo* study**

To assess test–retest reproducibility in healthy volunteers, we performed Bland–Altman agreement and reliability analyses. Bland–Altman analysis quantified the mean difference ($\mu_{diff}$) and limits of agreement (LoA). Reliability was assessed using the intraclass correlation coefficient (ICC) estimated with a two-way random-effects model. Variability was further summarized using coefficients of variation (CVs): Within-subject CV was defined as the test–retest standard deviation normalized by the overall mean; between-subject CV computed with each bundle across subjects and then averaged across bundles; and inter-bundle CV computed within each subject across bundles and then averaged across subjects.

These analyses were applied to ${RATIO}_{\mathrm{dosl}}$ and $T_{1D}$ maps (derived using analytical estimation and dictionary matching) with $B_{1}$ correction across 16 major white matter bundles. Figure S1.1 summarizes the in vivo test–retest results. For ${RATIO}_{\mathrm{dosl}}$, the bias was 0.001, the LoA ranged from −0.035 to 0.033, the ICC was 0.71 (95% confidence interval (CI): 0.62–0.78), the within-subject CV was 4.66%, the between-subject CV was 4.74%, and the inter-bundle CV was 7.67%. For $T_{1D}$ based on analytical estimation, the bias was 0.02 ms, the LoA ranged from −0.55 to 0.51 ms, the ICC was 0.70 (95% CI: 0.62–0.77), the within-subject CV was 4.27%, the between-subject CV was 4.09%, and the inter-bundle CV was 6.96%. For $T_{1D}$ based on dictionary matching, the bias was 0.022 ms, the LoA ranged from −0.59 to 0.55 ms, the ICC was 0.70 (95% CI: 0.61–0.77), the within-subject CV was 4.92%, the between-subject CV was 4.62%, and the inter-bundle CV was 7.96%.

Overall, ${RATIO}_{\mathrm{dosl}}$ and $T_{1D}$ exhibited negligible bias $\mu_{diff}$ and moderate-to-good reliability (ICC ≈ 0.70). The low within-subject CV (~4-5%) and between-subject CV (~4-5%) suggest reasonable test–retest precision and limited inter-individual variability in this healthy cohort. In contrast, the higher inter-bundle CV (~7–8%) indicates measurable bundle-dependent differences, consistent with anatomical contrast and potential sensitivity to bundle-specific microstructural variation. Further optimization is warranted to improve SNR and parameter sensitivity within hardware constraints, and to reduce scan time to minimize non-negligible motion (e.g., via fast 3D acquisitions), which may further enhance repeatability.


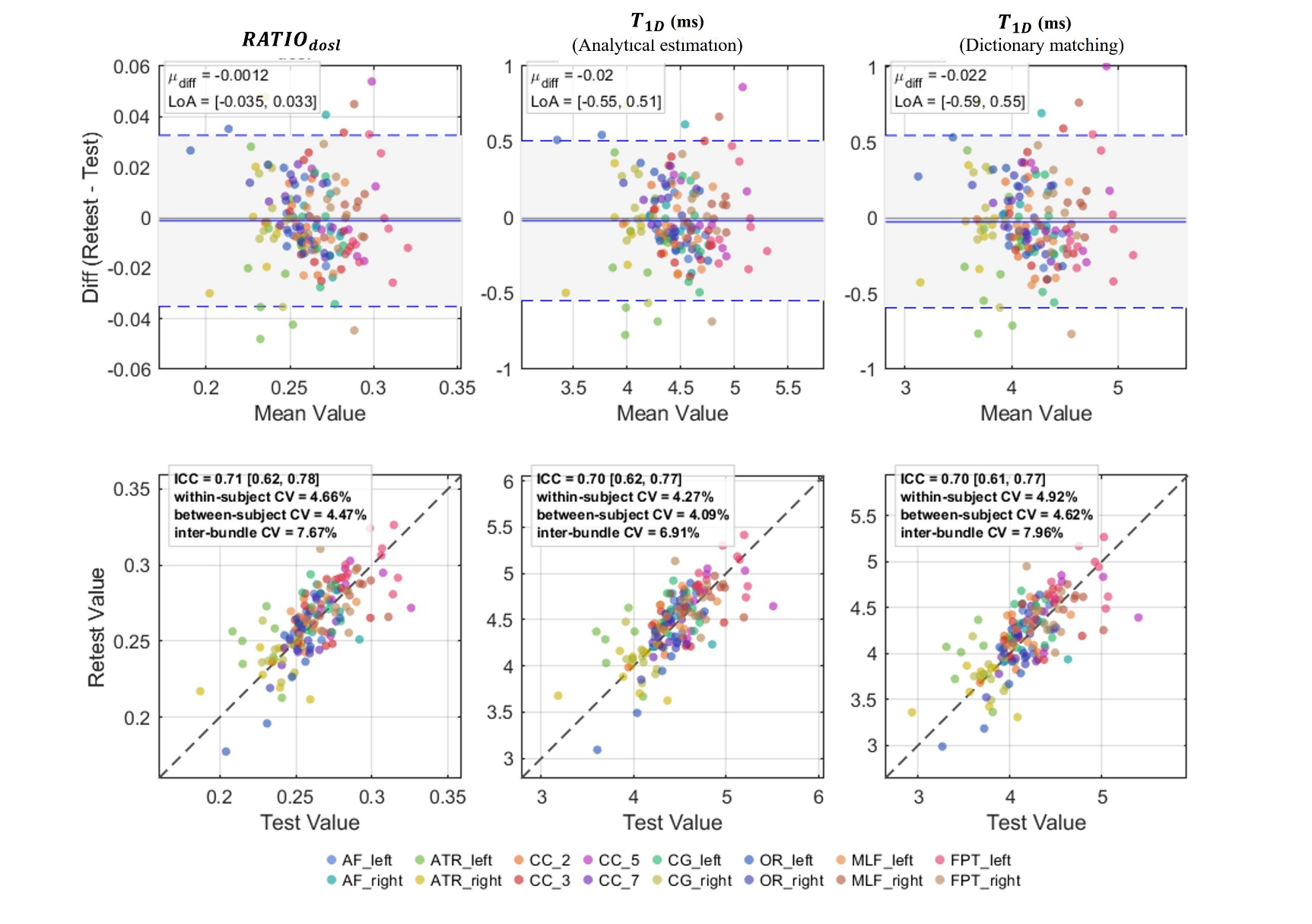


**Figure S1.1.** Bland–Altman plots and correlation plots for the human studies across the 16 major white matter fiber bundles in 10 volunteers.

# **3. The** $\boldsymbol{T}_{\boldsymbol{1}\boldsymbol{D}}$ **validation experiment**

To validate the obtained $T_{1D}$ values, we performed an additional *in vivo* experiment, in which $T_{s}$ of the dual-frequency spin-lock varied from 0.5 to 20 ms. As shown in Figure. S1.2, white matter exhibited enhanced contrast when $T_{s}\leq4$ms. Given the $T_{1D}$ filtering effect, this observation indirectly supports that the estimated $T_{1D}$ values (~3.70–4.80 ms) are reasonable.


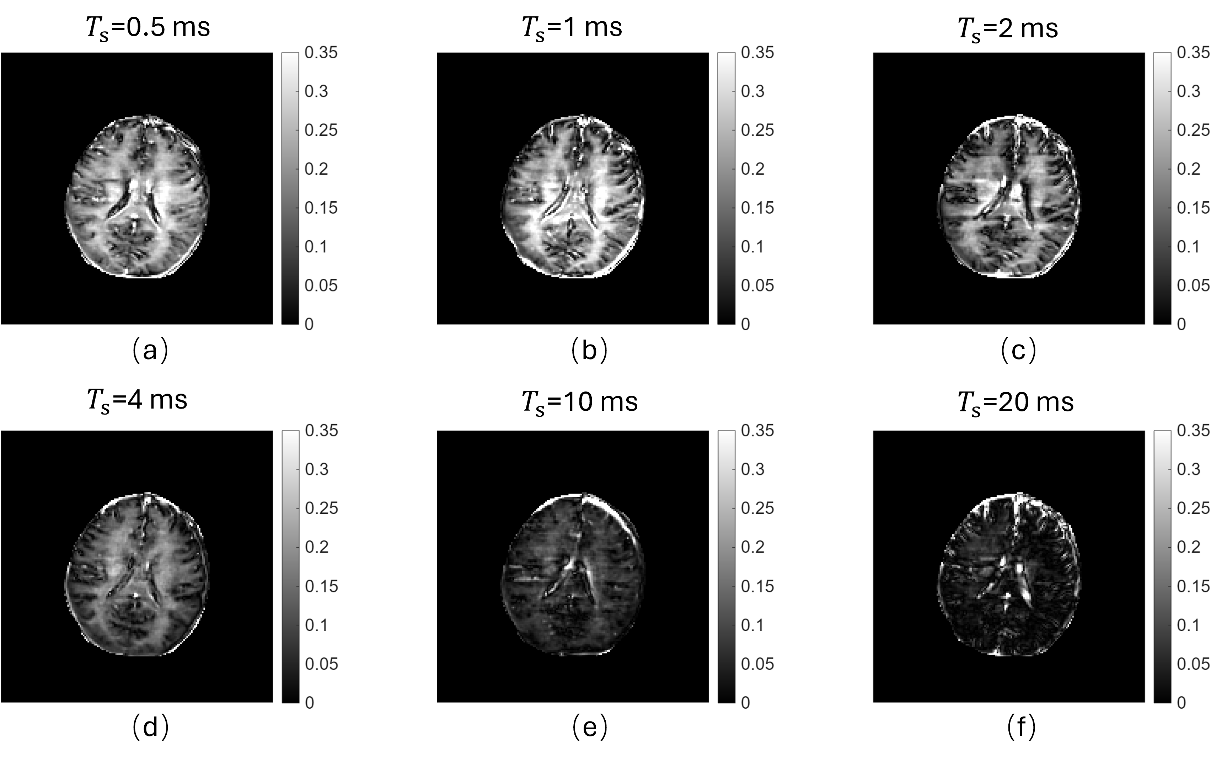


**Figure S1.2.** ${RATIO}_{dosl}$ maps acquired with dual-frequency spin-lock using $T_{s}$= 0.5, 1, 2, 4, 10, and 20 ms, while $T_{s}$ of the single-frequency spin-lock was fixed at 40 ms.

# **4. The influence of CEST-related effects**

To further assess potential contamination from CEST-related effects in the proposed $RATIO_{dosl}$, we performed an additional three-pool simulation including a free-water pool, a CEST pool, and an MT pool with dipolar order.

The simulation was performed using the same spin-lock framework as in Simulation study 1, with $\Delta\omega^{d\left( 1 \right)}/2\pi=5,6,7$ kHz and $\omega_{1}^{d\left( 1 \right)}/2\pi=500$ Hz. Here, $500$ Hz denotes the spin-lock amplitude (FSL), not the frequency offset. The baseline CEST parameters were set to $R_{2}^{CEST}=67$ Hz, $\Delta\omega_{CEST}=1.9$ ppm, $k_{CEST}=1500\text{ }s^{-1}$, and $f_{CEST}=0.14\%$. The sensitivity analysis was then performed over $R_{2}^{CEST}=30\text{–}75$ Hz, $k_{CEST}=1000\text{–}2500\text{ }s^{-1}$, and $f_{CEST}=0.1\%\text{–}1\%$.

Across these ranges, $RATIO_{dosl}$ exhibited only minor variation, as shown in Figure S1.3. For $\Delta\omega^{d\left( 1 \right)}/2\pi=5$ kHz (the setting used in this study), the relative changes were 0.042% (vs. $R_{2}^{CEST}$), 0.0003% (vs. $k_{CEST}$), and 0.112% (vs. $f_{CEST}$). These results demonstrate that, under the acquisition regime used in this work, CEST-related contamination is limited.


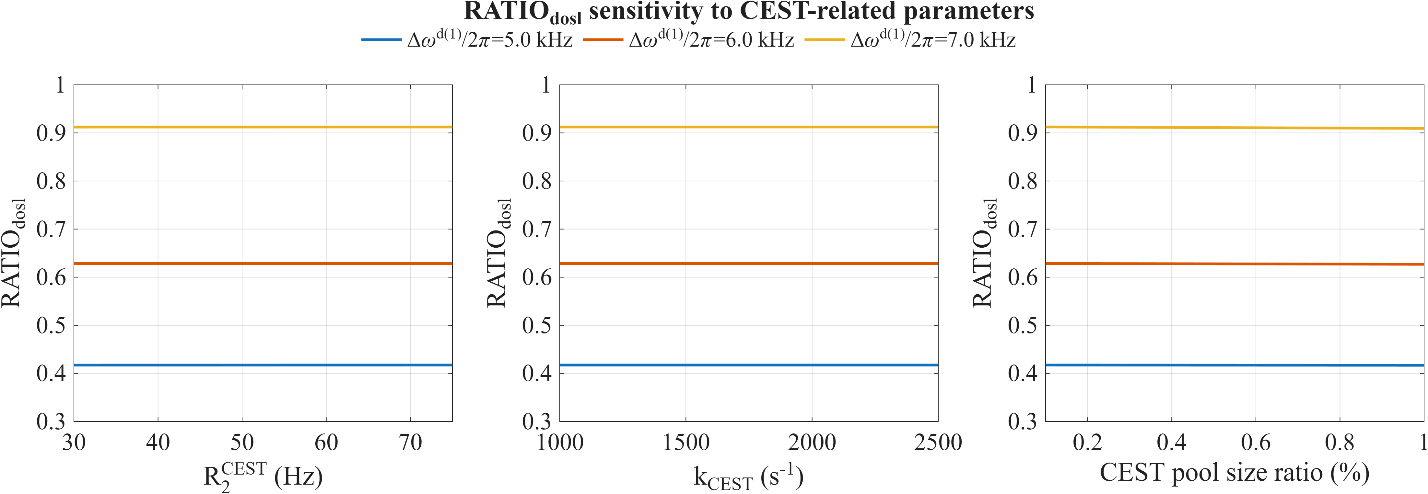


**Figure S1.3.** Sensitivity of $RATIO_{dosl}$ to CEST-related parameters in a three-pool simulation.
$RATIO_{dosl}$ is shown as a function of $R_{2}^{CEST}$, $k_{CEST}$, and CEST pool size ratio $f_{CEST}$, with curves corresponding to $\Delta\omega^{d\left( 1 \right)}/2\pi=5,6,7$ kHz. Baseline parameters were $R_{2}^{CEST}=67$ Hz, $\Delta\omega_{CEST}=1.9$ ppm, $k_{CEST}=1500\text{ }s^{-1}$, and $f_{CEST}=0.14\%$. Parameter ranges were $R_{2}^{CEST}=30\text{–}75$ Hz, $k_{CEST}=1000\text{–}2500\text{ }s^{-1}$, and $f_{CEST}=0.1\%\text{–}1\%$. The spin-lock amplitude was fixed at $\omega_{1}^{d\left( 1 \right)}/2\pi=500$ Hz (FSL).

# **5. The influence of** $\boldsymbol{T}_{\boldsymbol{2}\boldsymbol{b}}$ **for** $\boldsymbol{T}_{\boldsymbol{1}\boldsymbol{D}}$ **quantification**

To further investigate the bias in $T_{D}$ quantification, we conducted simulations to evaluate the sensitivity of the estimated $T_{1D}$ to $T_{2b}$. The tissue and spin-lock pulse parameters were chosen to be consistent with those used in the Simulation Studies section. As shown in Figure S1.4, varying $T_{2b}$ from 9 to 11 μs caused the estimated $T_{1D}$ to range from approximately 5 to 8 ms, compared with the ground truth value of 6.2 ms.


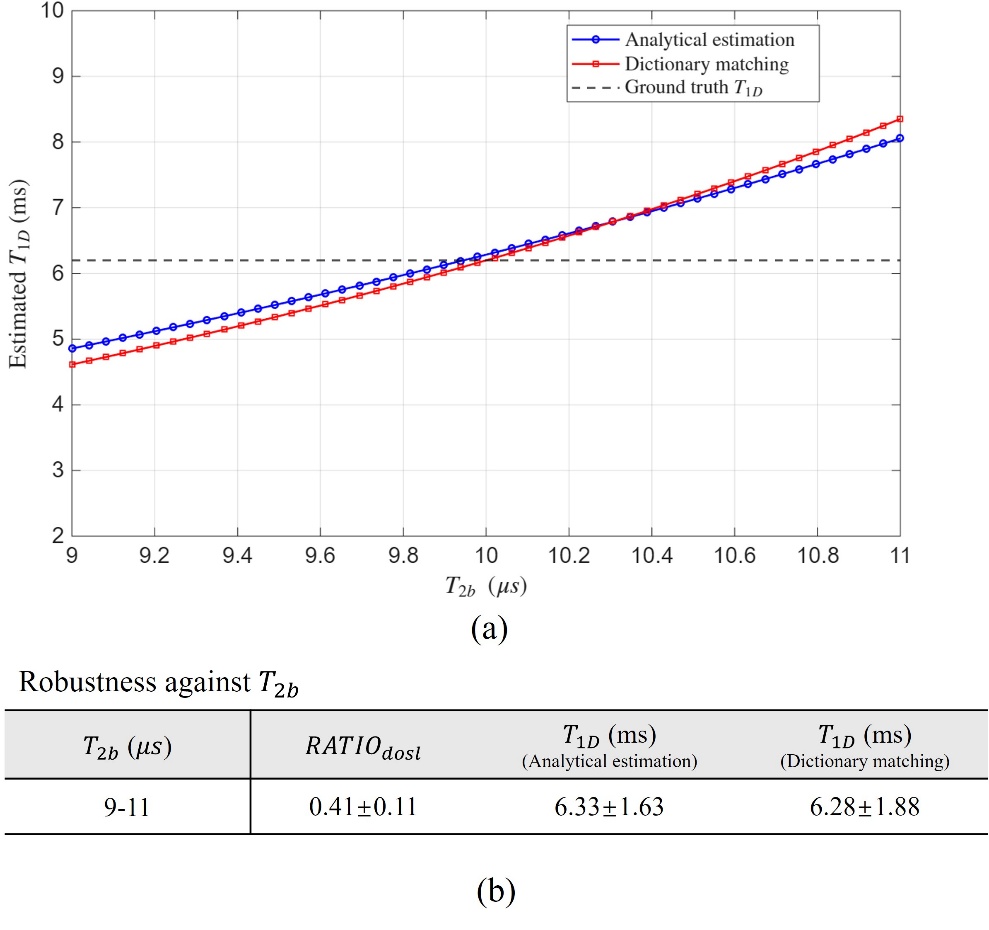


**Figure S1.4.** The sensitivity of estimated $T_{D}$ against $T_{2b}$ ranged from 9 to 11 $\mu s$.

**Reference**

1. Trott O, Palmer AG. R1ρ Relaxation outside of the Fast-Exchange Limit. *Journal of Magnetic Resonance*. 2002;154(1):157-160. doi:10.1006/jmre.2001.2466

2. Zaiss M, Zu Z, Xu J, et al. A combined analytical solution for chemical exchange saturation transfer and semi-solid magnetization transfer: AN ANALYTICAL SOLUTION FOR CEST AND MT. *NMR Biomed*. 2015;28(2):217-230. doi:10.1002/nbm.3237

3. Hou J, Wong VW, Jiang B, et al. Macromolecular proton fraction mapping based on spin‐lock magnetic resonance imaging. *Magn Reson Med*. 2020;84(6):3157-3171. doi:10.1002/mrm.28362
