# Supplementary material for: Dipolar Order Mapping Based on Spin‐Lock Magnetic Resonance Imaging
Source: NMR Biomed. 2026 Jun 17;39(7):e70331. doi: 10.1002/nbm.70331 (PMC13275185; doi:10.1002/nbm.70331)
Supplement: Supplementary file 2 — Figure S2:1. MPF map, RATIOdosl map, and T1D maps (derived using analytical estimation and dictionary matching) with B1 correction in volunteer V1. Figure S2:2. MPF map, RATIOdosl map, and T1D maps (derived using analytical estimation and dictionary matching) with B1 correction in the V1 retest. Figure S2:3. MPF map, RATIOdosl map, and T1D maps (derived using analytical estimation and dictionary matching) with B1 correction in volunteer V2. Figure S2:4. MPF map, RATIOdosl map, and T1D maps (derived using analytical estimation and dictionary matching) with B1 correction in the V2 retest. Figure S2:5. MPF map, RATIOdosl map, and T1D maps (derived using analytical estimation and dictionary matching) with B1 correction in volunteer V3. Figure S2:6. MPF map, RATIOdosl map, and T1D maps (derived using analytical estimation and dictionary matching) with B1 correction in the V3 retest. Figure S2:7. MPF map, RATIOdosl map, and T1D maps (derived using analytical estimation and dictionary matching) with B1 correction in volunteer V4. Figure S2:8. MPF map, RATIOdosl map, and T1D maps (derived using analytical estimation and dictionary matching) with B1 correction in the V4 retest. Figure S2:9. MPF map, RATIOdosl map, and T1D maps (derived using analytical estimation and dictionary matching) with B1 correction in volunteer V5. Figure S2:10. MPF map, RATIOdosl map, and T1D maps (derived using analytical estimation and dictionary matching) with B1 correction in the V5 retest. Figure S2:11. MPF map, RATIOdosl map, and T1D maps (derived using analytical estimation and dictionary matching) with B1 correction in volunteer V6. Figure S2:12. MPF map, RATIOdosl map, and T1D maps (derived using analytical estimation and dictionary matching) with B1 correction in the V6 retest. Figure S2:13. MPF map, RATIOdosl map, and T1D maps (derived using analytical estimation and dictionary matching) with B1 correction in volunteer V7. Figure S2:14. MPF map, RATIOdosl map, and T1D maps (derived using [file NBM-39-e70331-s002.docx]

# **Supporting Information 2**


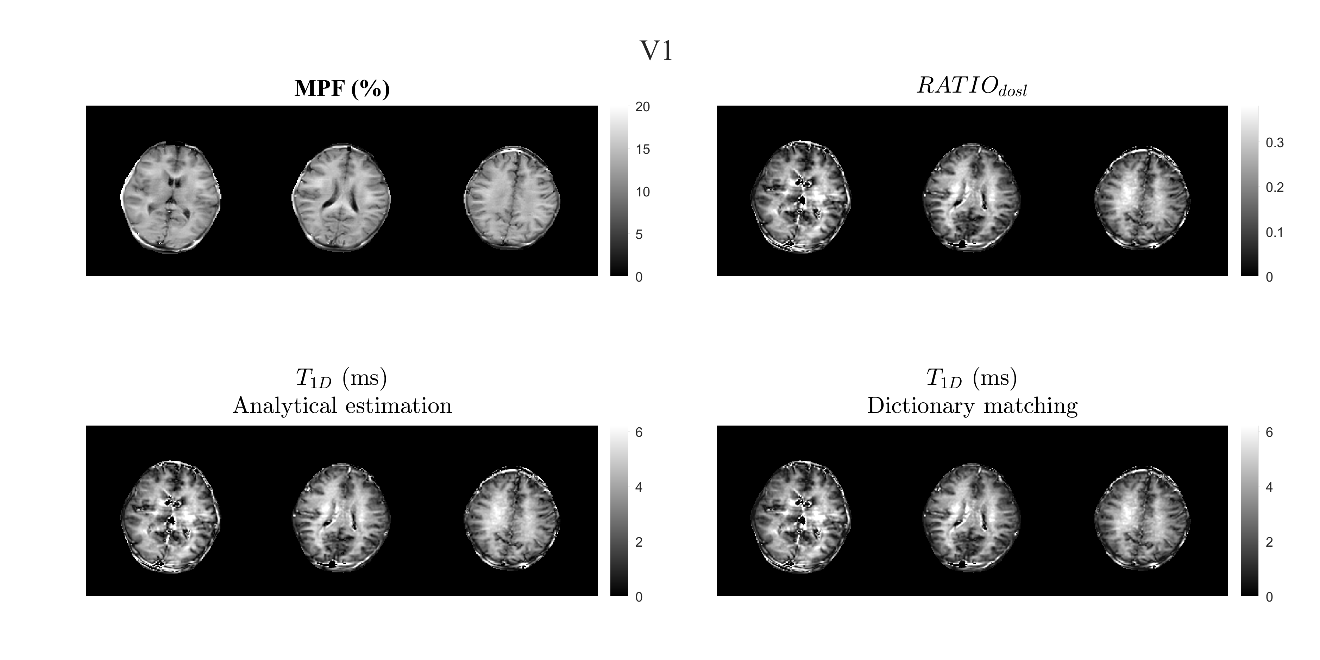


**Figure S2.1.** MPF map, ${RATIO}_{dosl}$ map, and $T_{1D}$maps (derived using analytical estimation and dictionary matching) with $B_{1}$correction in volunteer V1.
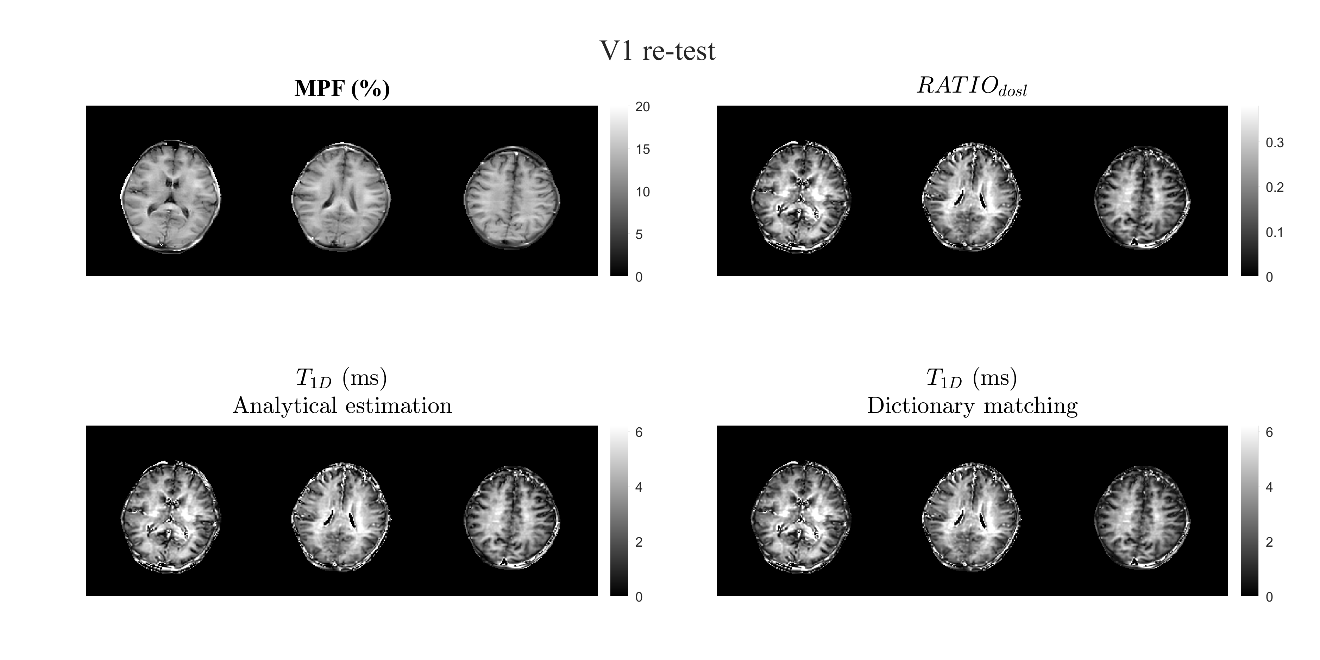


**Figure S2.2.** MPF map, ${RATIO}_{dosl}$ map, and $T_{1D}$maps (derived using analytical estimation and dictionary matching) with $B_{1}$ correction in the V1 re-test.


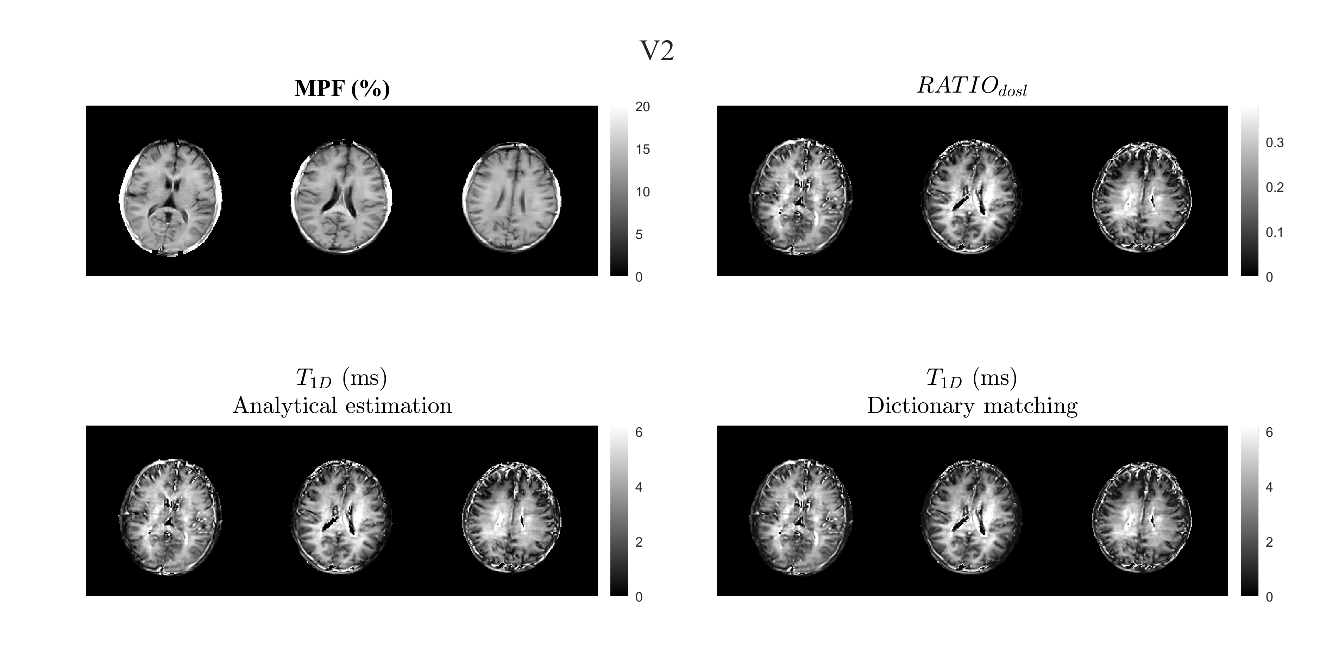


**Figure S2.3.** MPF map, ${RATIO}_{dosl}$map, and $T_{1D}$maps (derived using analytical estimation and dictionary matching) with $B_{1}$correction in volunteer V2.
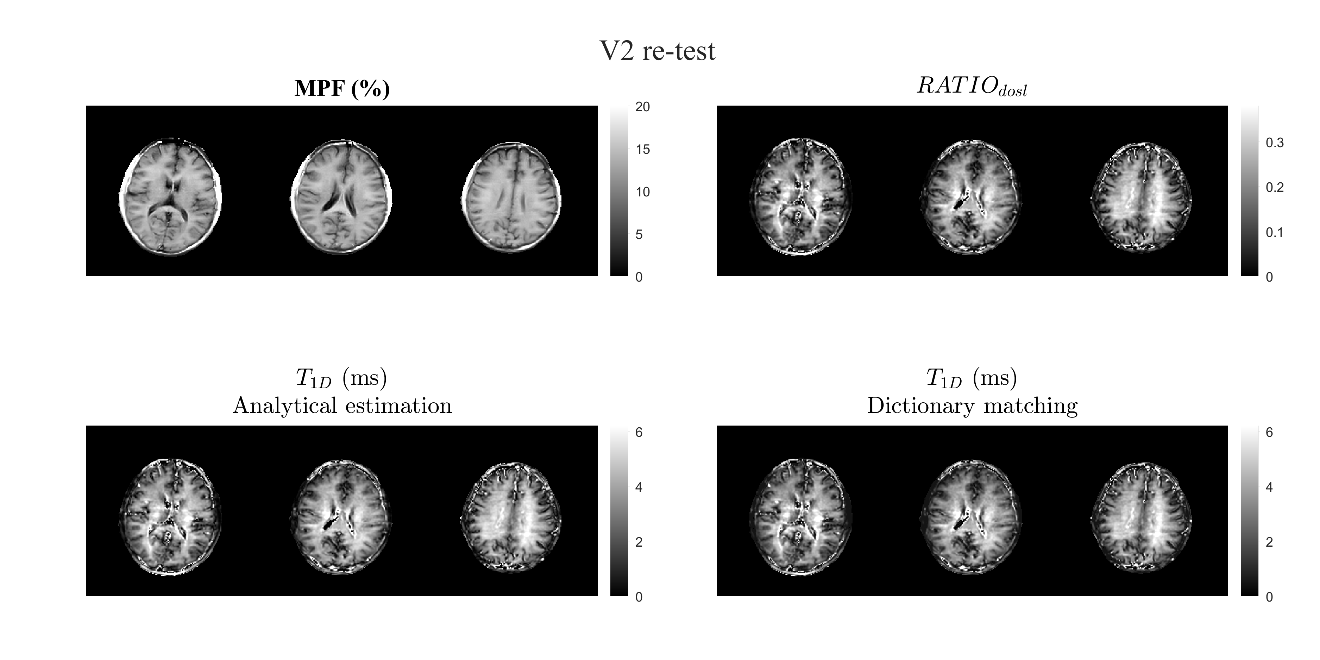


**Figure S2.4.** MPF map, ${RATIO}_{dosl}$ map, and $T_{1D}$maps (derived using analytical estimation and dictionary matching) with $B_{1}$ correction in the V2 re-test.


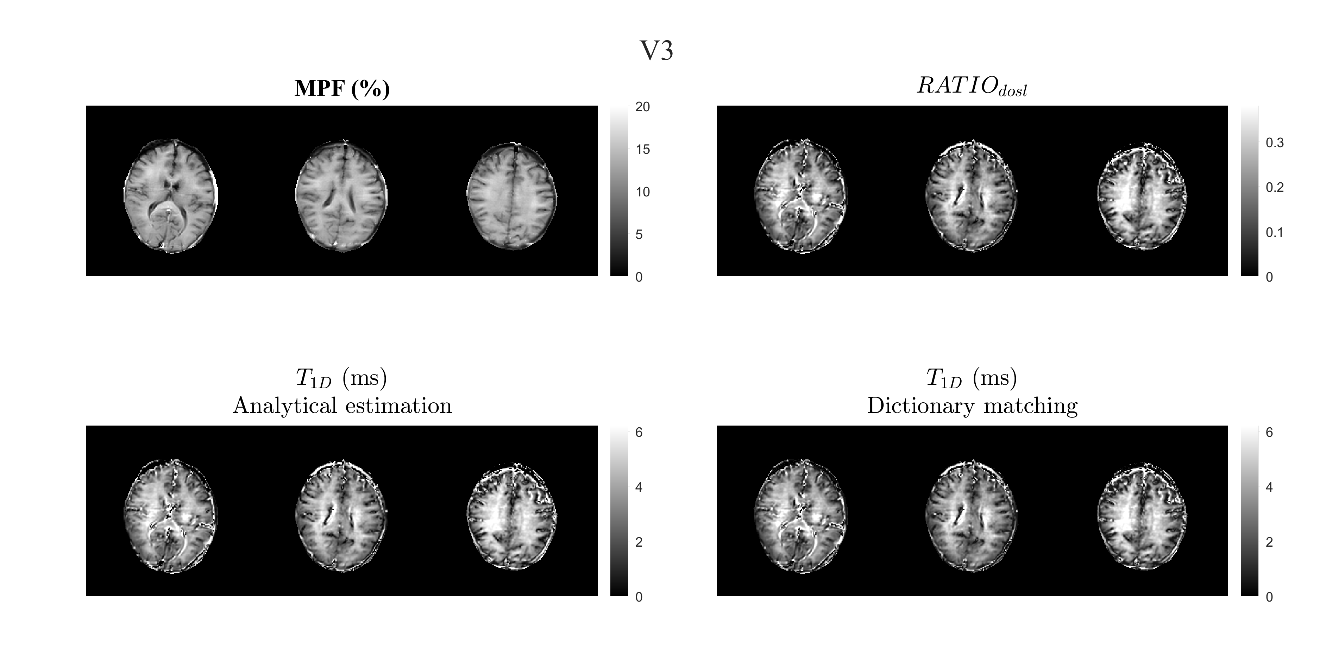


**Figure S2.5.** MPF map, ${RATIO}_{dosl}$ map, and $T_{1D}$ maps (derived using analytical estimation and dictionary matching) with $B_{1}$ correction in volunteer V3.
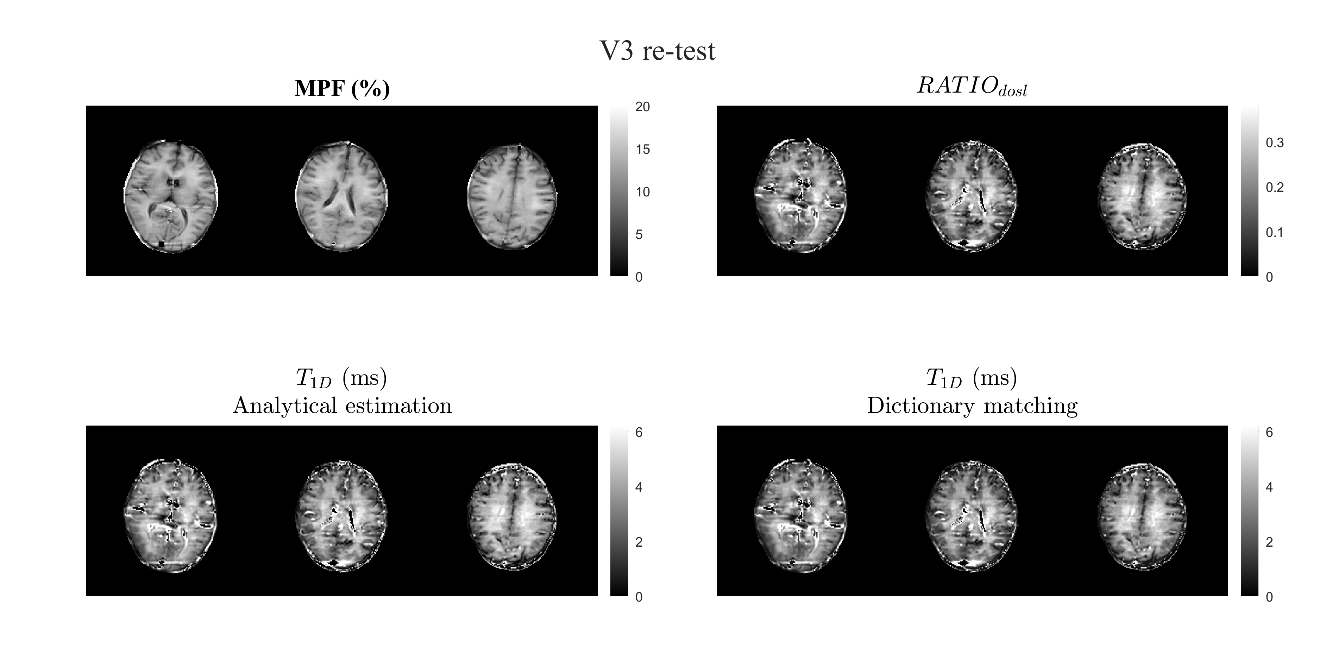


**Figure S2.6.** MPF map, ${RATIO}_{dosl}$map, and $T_{1D}$maps (derived using analytical estimation and dictionary matching) with $B_{1}$correction in the V3 re-test.


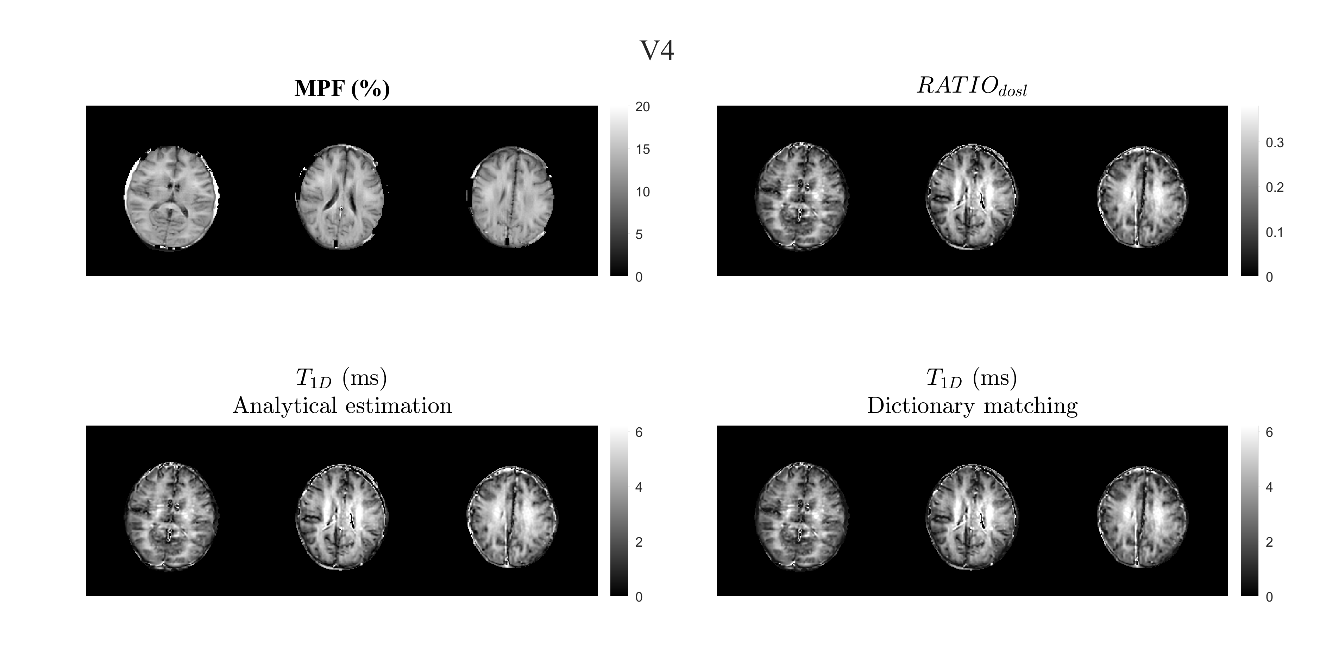


**Figure S2.7.** MPF map, ${RATIO}_{dosl}$ map, and $T_{1D}$maps (derived using analytical estimation and dictionary matching) with $B_{1}$ correction in volunteer V4.
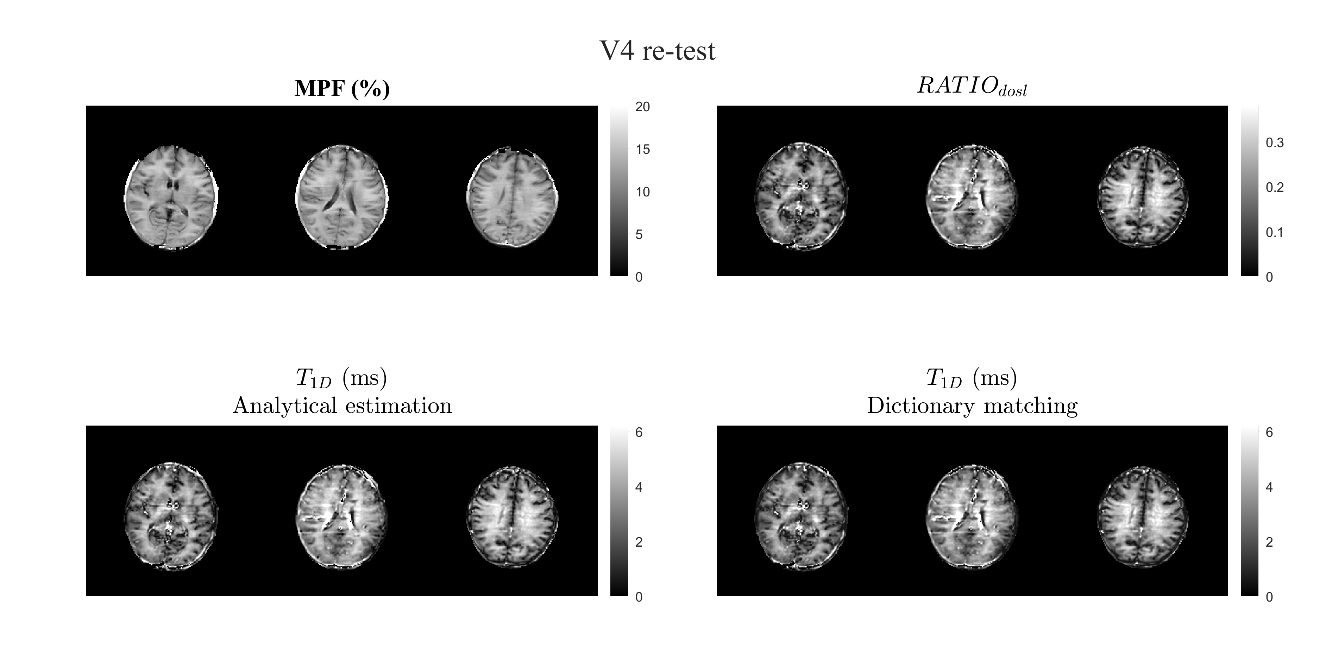


**Figure S2.8.** MPF map, ${RATIO}_{dosl}$ map, and $T_{1D}$ maps (derived using analytical estimation and dictionary matching) with $B_{1}$ correction in the V4 re-test.


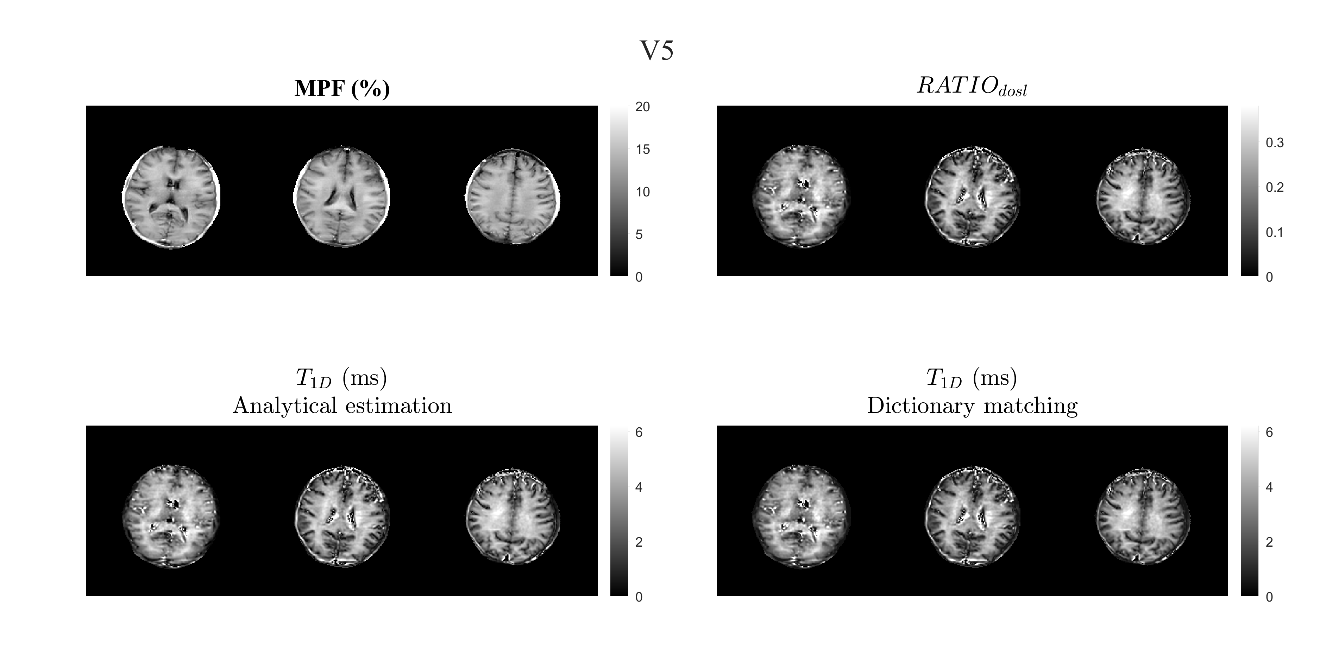


**Figure S2.9.** MPF map, ${RATIO}_{dosl}$ map, and $T_{1D}$ maps (derived using analytical estimation and dictionary matching) with $B_{1}$correction in volunteer V5.
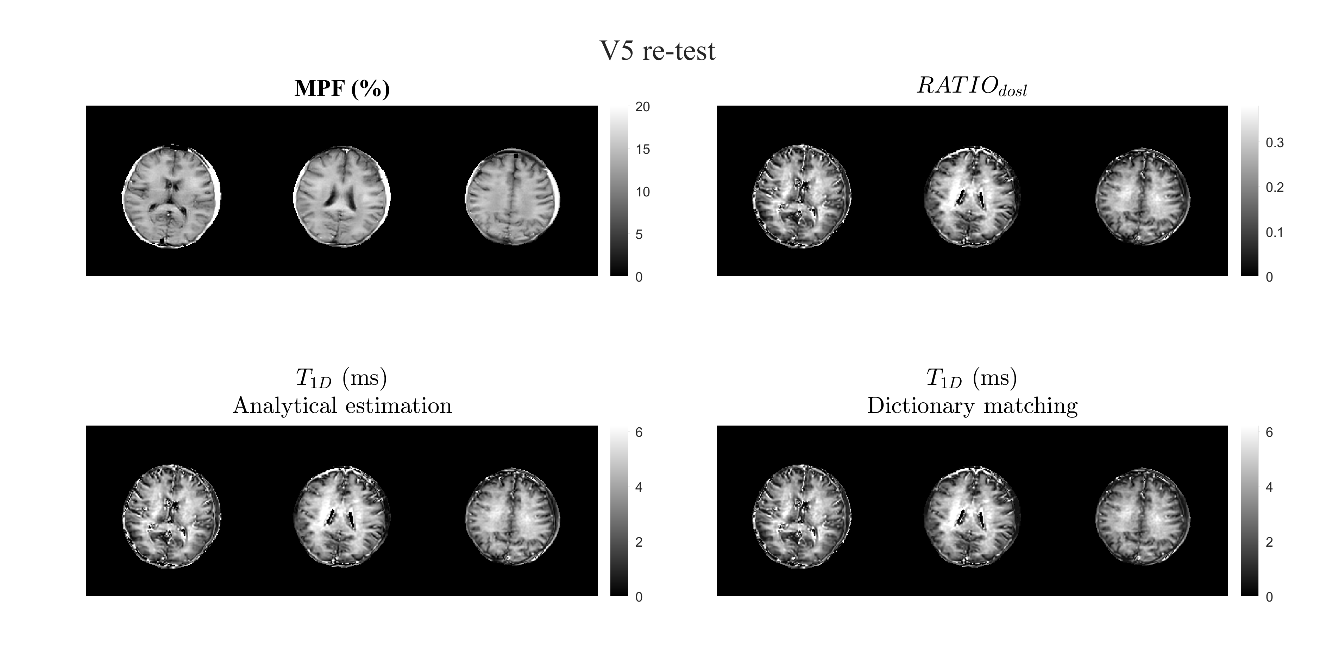


**Figure S2.10.** MPF map, ${RATIO}_{dosl}$ map, and $T_{1D}$ maps (derived using analytical estimation and dictionary matching) with $B_{1}$ correction in the V5 re-test.


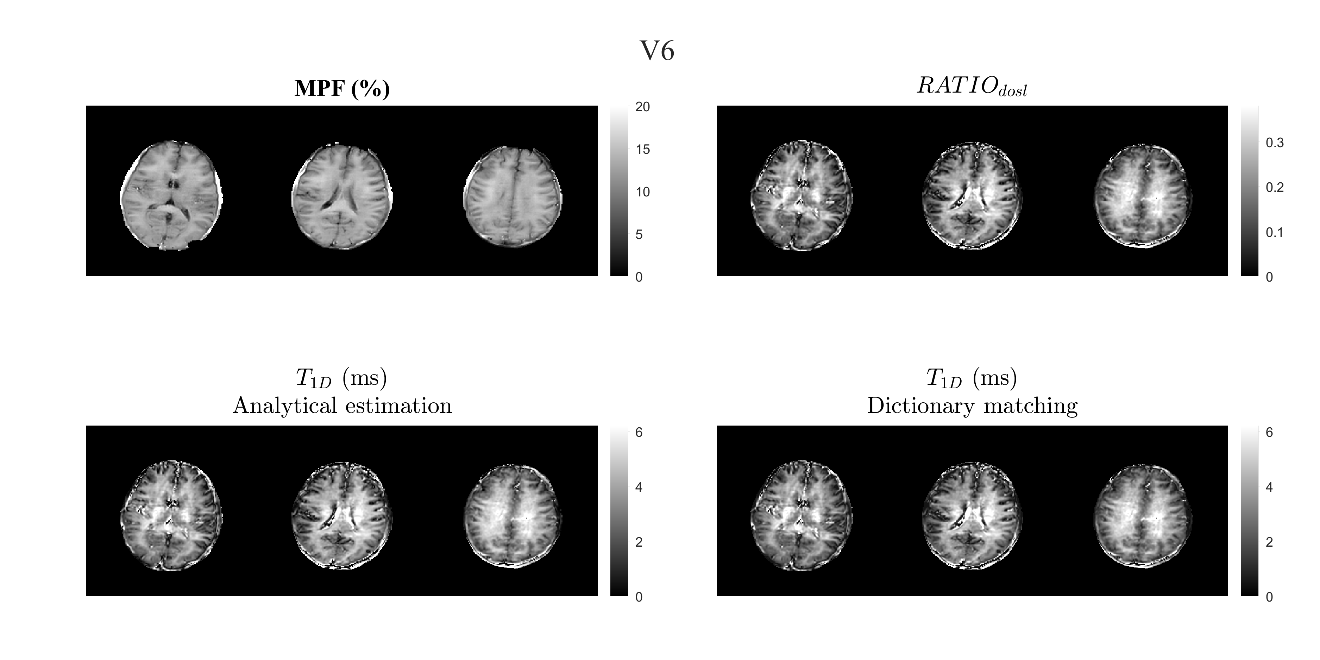


**Figure S2.11.** MPF map, ${RATIO}_{dosl}$map, and $T_{1D}$maps (derived using analytical estimation and dictionary matching) with $B_{1}$correction in volunteer V6.
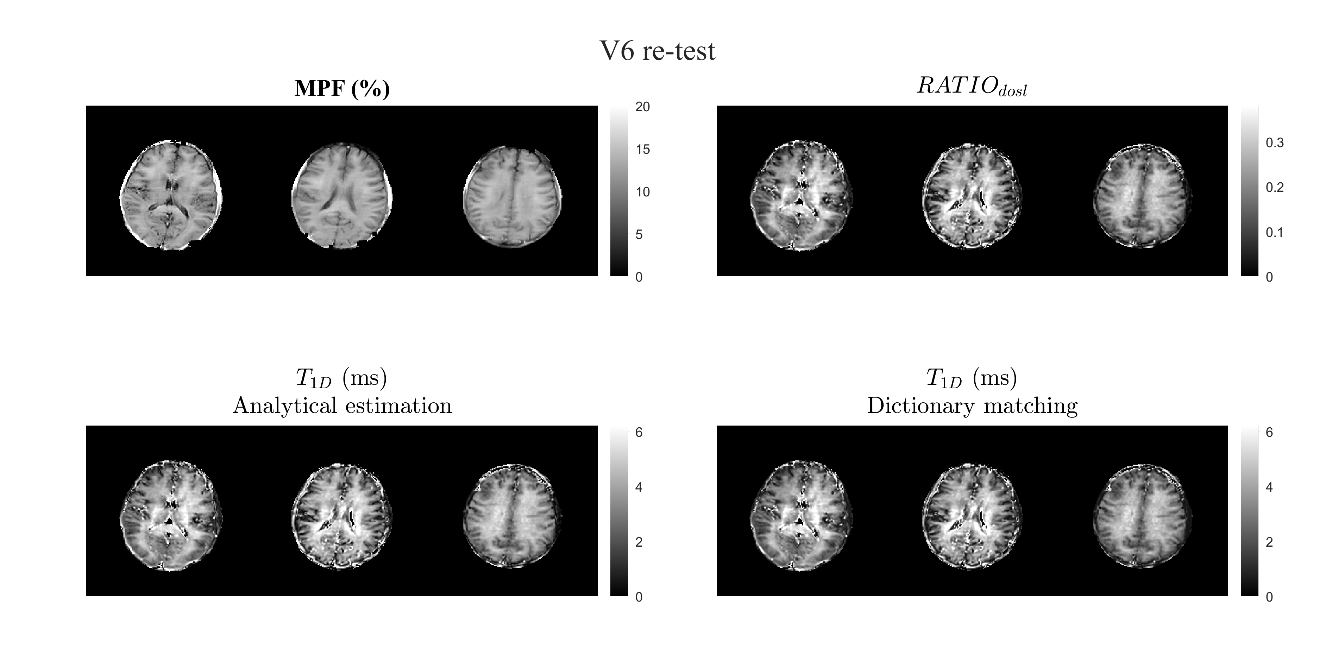


**Figure S2.12.** MPF map, ${RATIO}_{dosl}$ map, and $T_{1D}$ maps (derived using analytical estimation and dictionary matching) with $B_{1}$correction in the V6 re-test.


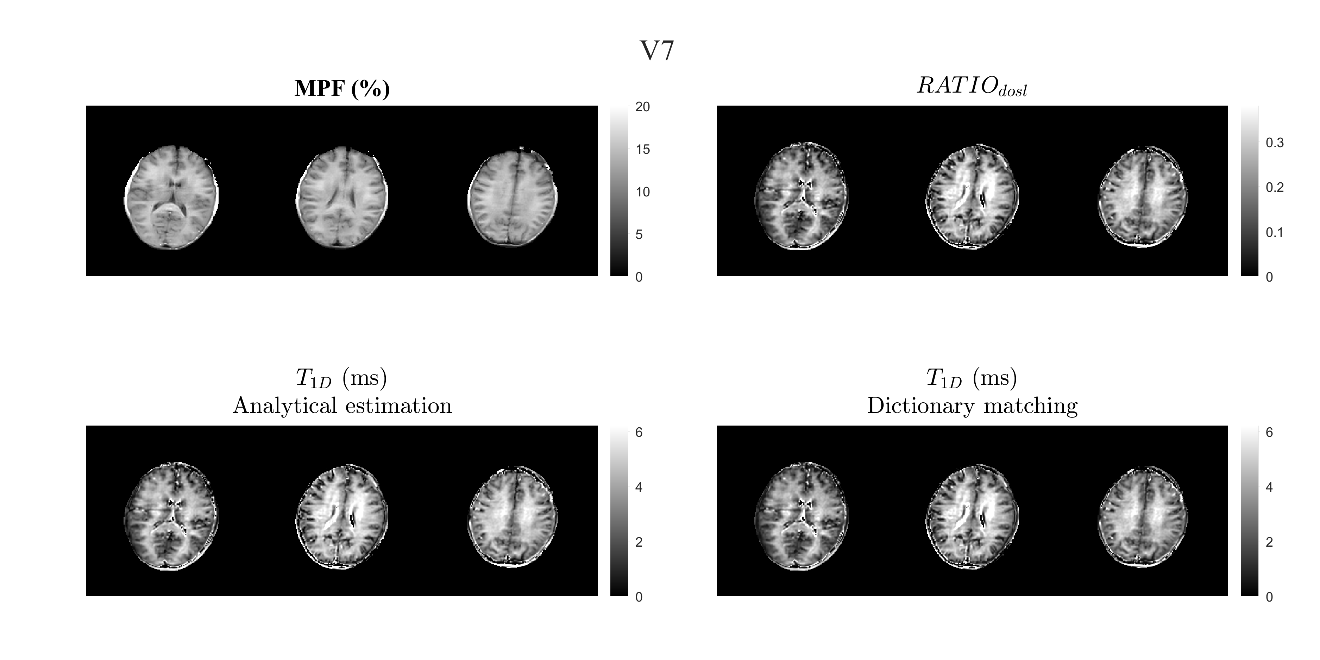


**Figure S2.13.** MPF map, ${RATIO}_{dosl}$map, and $T_{1D}$maps (derived using analytical estimation and dictionary matching) with $B_{1}$correction in volunteer V7.
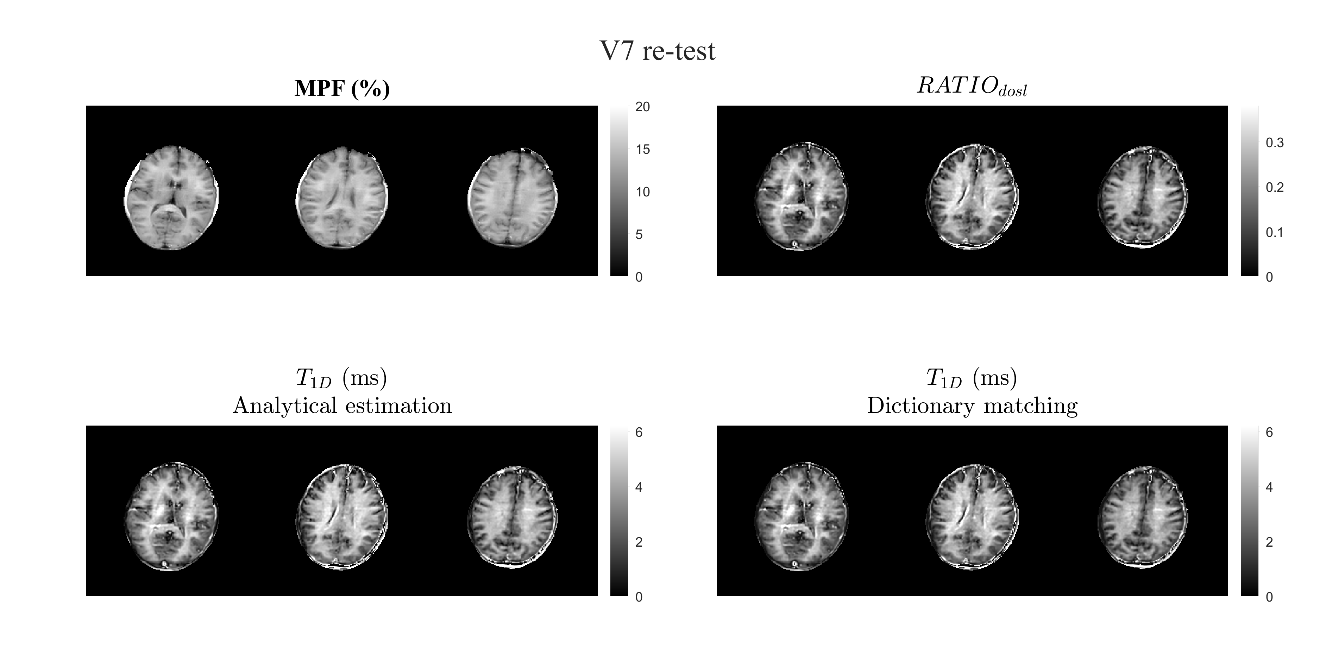


**Figure S2.14.** MPF map, ${RATIO}_{dosl}$ map, and $T_{1D}$ maps (derived using analytical estimation and dictionary matching) with $B_{1}$ correction in the V7 re-test.


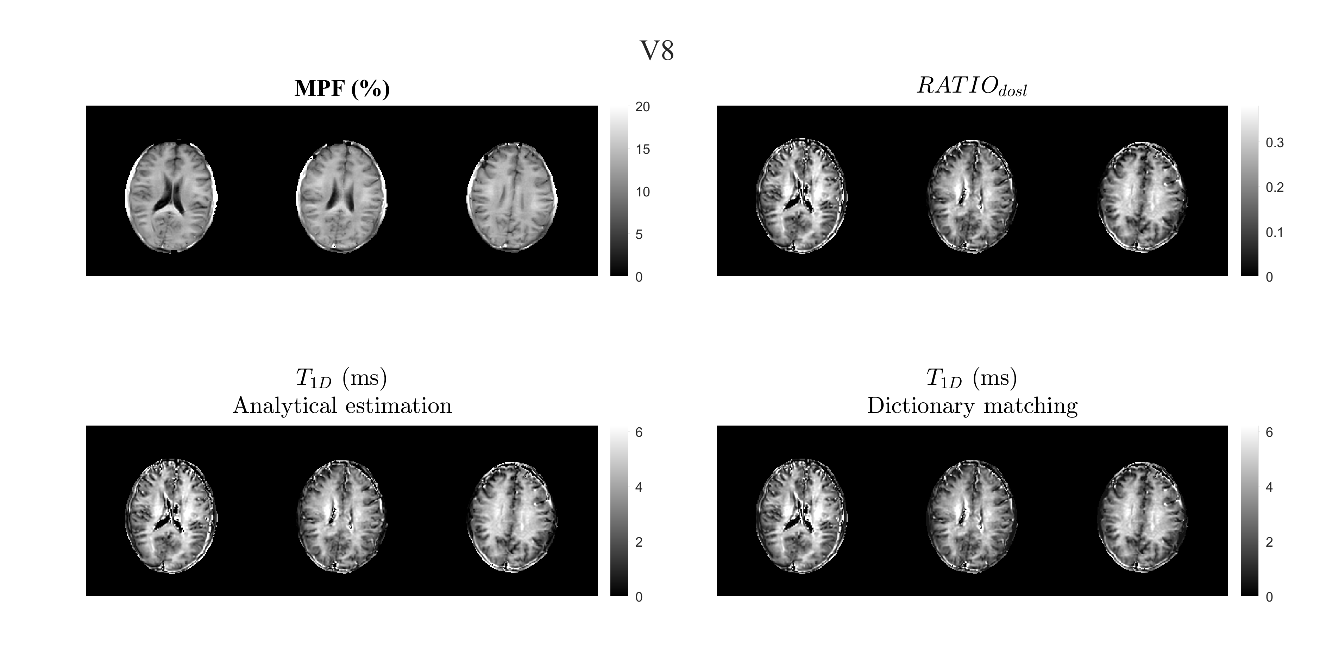


**Figure S2.15.** MPF map, ${RATIO}_{dosl}$map, and $T_{1D}$maps (derived using analytical estimation and dictionary matching) with $B_{1}$correction in volunteer V8.
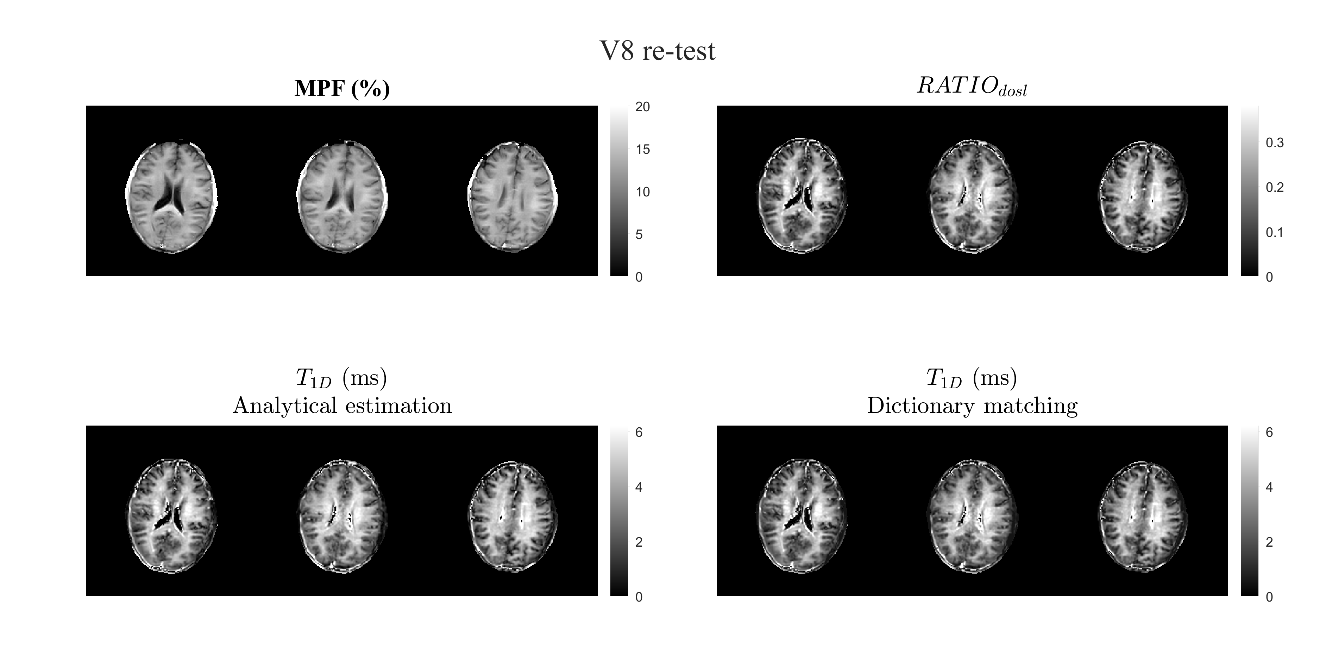


**Figure S2.16.** MPF map, ${RATIO}_{dosl}$ map, and $T_{1D}$ maps (derived using analytical estimation and dictionary matching) with $B_{1}$ correction in the V8 re-test.


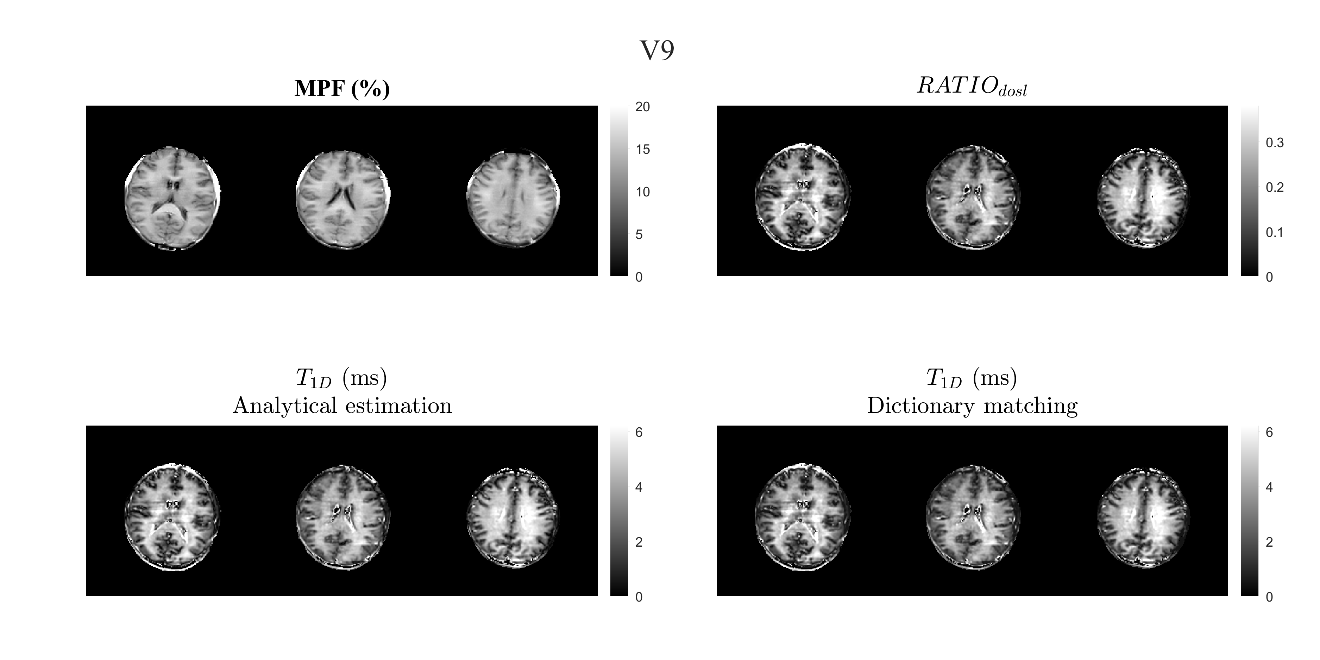


**Figure S2.17.** MPF map, ${RATIO}_{dosl}$map, and $T_{1D}$maps (derived using analytical estimation and dictionary matching) with $B_{1}$correction in volunteer V9.
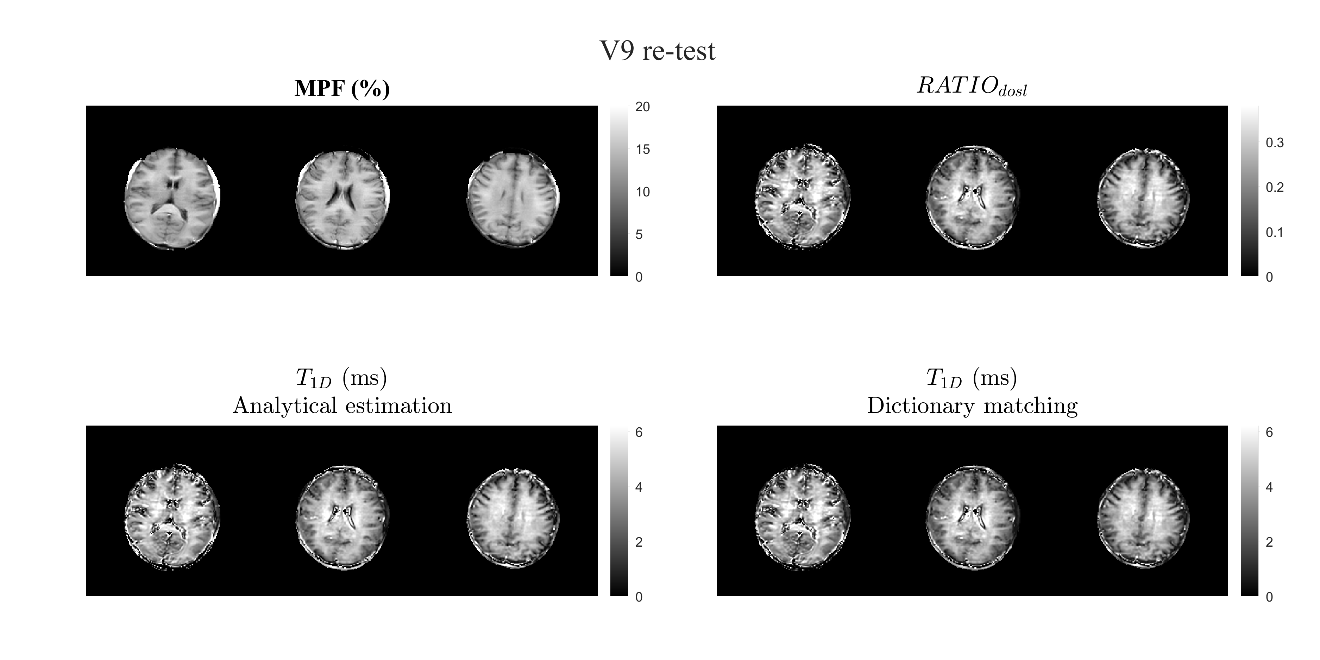


**Figure S2.18.** MPF map, ${RATIO}_{dosl}$ map, and $T_{1D}$ maps (derived using analytical estimation and dictionary matching) with $B_{1}$ correction in the V9 re-test.


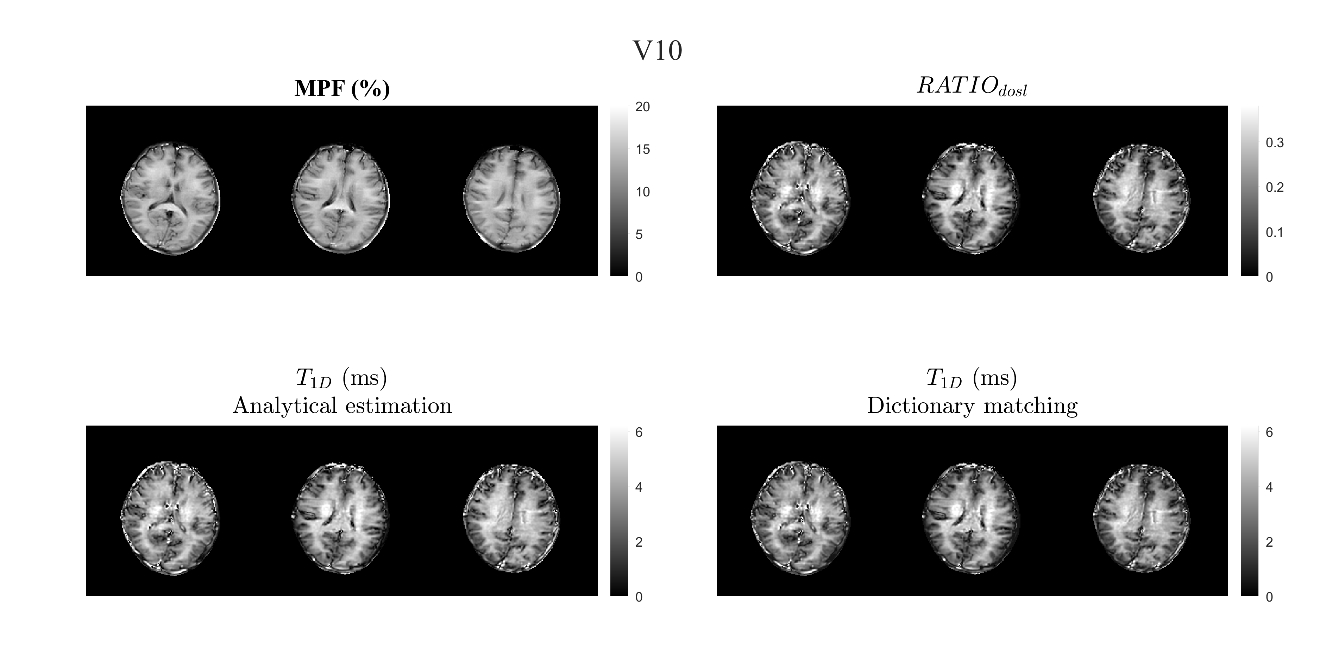


**Figure S2.19.** MPF map, ${RATIO}_{dosl}$ map, and $T_{1D}$ maps (derived using analytical estimation and dictionary matching) with $B_{1}$ correction in volunteer V10.
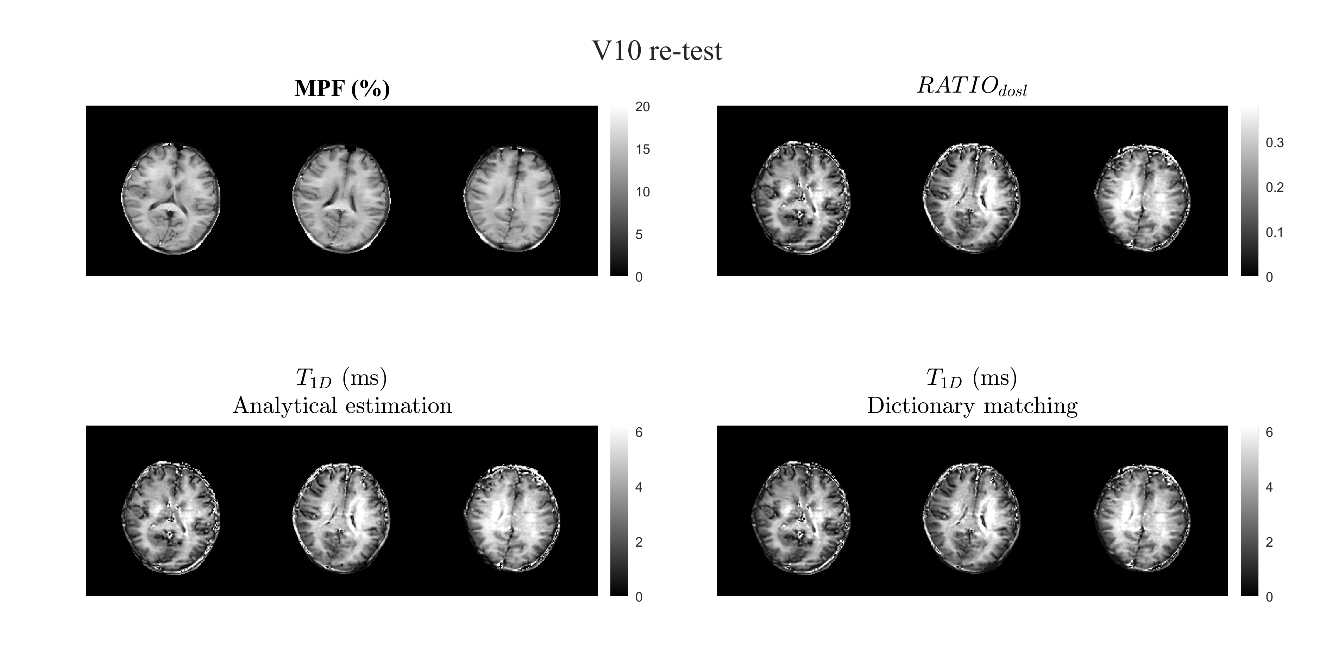


**Figure S2.20.** MPF map, ${RATIO}_{dosl}$ map, and $T_{1D}$ maps (derived using analytical estimation and dictionary matching) with $B_{1}$ correction in the V10 re-test.
